# Minimal intervening control of biomolecular networks leading to a desired cellular state

**DOI:** 10.1038/s41598-019-49571-6

**Published:** 2019-09-11

**Authors:** Sang-Mok Choo, Sang-Min Park, Kwang-Hyun Cho

**Affiliations:** 1 Department of Mathematics, University of Ulsan, Ulsan, 44610 Republic of Korea; 2 Department of Bio and Brain Engineering, Korea Advanced Institute of Science and Technology (KAIST), Daejeon, 34141 Republic of Korea

**Keywords:** Dynamic networks, Control theory

## Abstract

A cell phenotype can be represented by an attractor state of the underlying molecular regulatory network, to which other network states eventually converge. Here, the set of states converging to each attractor is called its basin of attraction. A central question is how to drive a particular cell state toward a desired attractor with minimal interventions on the network system. We develop a general control framework of complex Boolean networks to provide an answer to this question by identifying control targets on which one-time temporary perturbation can induce a state transition to the boundary of a desired attractor basin. Examples are shown to illustrate the proposed control framework which is also applicable to other types of complex Boolean networks.

## Introduction

Various complex phenomena across different disciplines are often explained by the complicated interactions of their constituting elements, resulting in a class of models called *complex networks*^1–4^. In the study of complex networks, our ultimate goal is to control their steady state behavior such that a desired behavior is achieved^5^. For instance, in cancer study, we want to identify target molecule(s) in the complex intracellular molecular regulatory network that can effectively induce apoptosis of cancer cells, where the desired steady state behavior is apoptosis represented by a particular converging steady state of molecules, called a *desired attractor*^6^.

A number of studies have been conducted to control complex networks and to achieve a desired behavior, but most of them assumed persistent perturbation of the identified target elements^7–11^. Such approaches, however, have critical limitations if the duration of perturbation is difficult to be specified in advance or persistence perturbation might cause other significant problems as in the case of biomedical applications where long-term treatment of drugs might cause resistance or side-effects^12^. So, in this study, we present a different kind of Boolean network control based on temporary perturbation instead of persistent perturbation. Moreover, to achieve minimal intervention, our control strategy drives any initial state of a complex Boolean network only to the boundary of basin of attraction to the desired attractor where a state in the basin autonomously converges to the corresponding attractor eventually. This boundary-reaching control (BRC) can ensure to achieve the desired steady state behavior of any complex Boolean network by only temporarily perturbing the identified minimal number of control target nodes. To implement the BRC, we need to find out the exact basin of attraction of a given desired attractor and to further delineate the boundary of a basin with respect to the current initial state of a network. There is, however, a computational challenge in identifying such an exact basin due to the computational complexity that increases exponentially with the network size^13^. Here we present an algorithm to identify the exact basin of attraction using the topological property as well as regulatory logics of complex networks. We also present an algorithm to identify the boundary of a basin by measuring the distance between the current state and the basin in the state space. We can then identify control targets for BRC from the information of boundary. Our BRC is applicable irrespective of the relative size of basin of attraction while it becomes easier to identify control targets when the basin of a desired attractor is large enough.

We illustrate the BRC using toy example networks and demonstrate its usefulness by applying it to biomolecular regulatory networks of different scales. We further show that it can also be applied to other types of complex networks such as an ecological network and an insect social network. The BRC is a generic control strategy applicable to any complex Boolean networks and it ensures the convergence of any network state to the desired attractor through minimal and temporary intervention of network dynamics.

## Results

### Overview of boundary-reaching control

**Figure 1:**
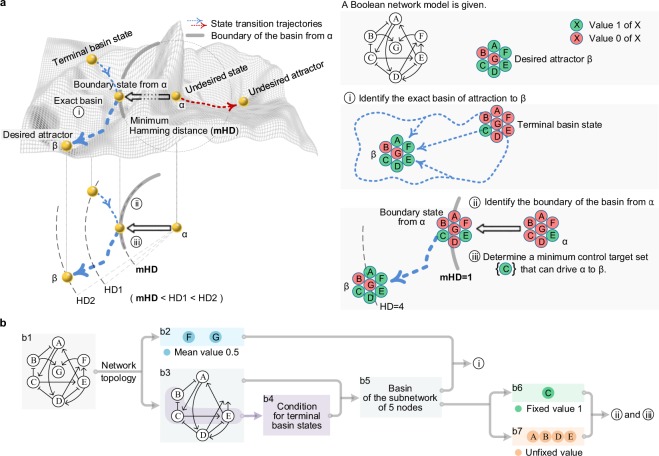
Overview of BRC. (**a**) The overall flow of BRC process is illustrated in three steps. Given a Boolean network model (top right: arrows and bar-headed lines represent activation and inhibition, respectively) and a desired attractor state β (A, B, C, D, E, F, G) = (1, 0, 1, 1, 1, 1, 0), the first step $\overline{)\,{\rm{i}}\,}$ (middle right) is to identify the exact basin of β in a hierarchical and algebraic way, searching from β to the terminal states of state transition trajectories. So, the exact basin contains those states upon the state transition trajectory from the terminal state (0, 0, 1, 0, 0, 0, 0) to β, represented by two connected dotted blue arrows on the attractor landscape (left). The second step $\overline{)\,{\rm{ii}}\,}$ is to identify the boundary of the basin from an undesired state α converging to an undesired attractor where the convergence and the boundary are represented by the blue dotted arrow and the thick arc of a dotted circle in top left. The boundary consists of those states of having a minimum Hamming distance from α. Bottom left shows that a boundary state might not be a terminal state. The last step $\overline{){\rm{iii}}}$ is to determine a boundary state to which α is to be driven and then a minimum control target set is determined, where all the leftward arrows denote driving α (0, 0, 0, 0, 1, 0, 0) to the boundary state (0, 0, 1, 0, 1, 0, 0) and thereby {C = 0} becomes the minimum control target set (bottom right). (**b**) The overall flow of the three steps is summarized. The first step $\overline{)\,{\rm{i}}\,}$ begins to divide the model (b1) into symmetric nodes F and G (b2) and a subnetwork of the remaining five nodes (b3). The topological and algebraic structures (B, C and E in purple) provide conditions for terminal states (b4), which are used to identify the basin of the reduced attractor β′ (A, B, C, D, E) = (1, 0, 1, 1, 1) in the subnetwork (b5). Concatenation of each basin state of β′ and all possible states of (F, G) results in the exact basin of β. The basin of β′ is decomposed into two collections b6 and b7 for $\overline{)\,{\rm{ii}}\,}$ and $\overline{){\rm{iii}}}$. See Results, Supplementary Figs 1–3 and Methods for details.

The BRC can be summarized in three steps as shown in Fig. 1a. Let us consider a Boolean network model in Fig. 1a where its state update logics are given (Supplementary Fig. 1) and its current state is at an undesired state α which will eventually converge to an undesired attractor. Since BRC is a control strategy to drive the undesired state to a state in the basin of a desired attractor β in Fig. 1a by perturbing a minimum number of nodes, we first need to identify the exact basin of the desired attractor and search a minimum set of target nodes (first step $\overline{)\,{\rm{i}}\,}$ in Fig. 1a). Each basin state is hierarchically identified in a sequential manner: the desired attractor state is located at the 1^st^ layer. A given state *S*_ℓ_ at the ℓ^th^ layer (ℓ ≥ 1) is substituted in the state at time step *t* + 1 in the Boolean update rules to get a system of equations and the solution states *S*_ℓ+1_ are located at the (ℓ + 1)^th^ layer, where the given state is called a terminal basin state if no more solution state is calculated. The first step is completed when every terminal basin state is identified. The boundary concept of the basin (second step $\overline{)\,{\rm{ii}}\,}$ in Fig. 1a) is related to a basin state to which α is driven to transit by perturbing (i.e. changing) some nodes’ values in α, where these nodes are called “control target nodes” and the number of these nodes is a “Hamming distance (HD) from α to the basin state”. In particular, a minimum set of control target nodes with their target values is referred as a “minimum control target set”. A basin state is called a “boundary state from α” if the basin state is located away from α with the minimum HD (mHD), and the set of boundary states from α is referred to as the “boundary of the basin from α”. For instance, in the right of Fig. 1a, the HD between the basin state (A, B, C, D, E, F, G) = (0, 0, 1, 0, 1, 0, 0) and α (0, 0, 0, 0, 1, 0, 0) is equal to 1 and therefore (0, 0, 1, 0, 1, 0, 0) is a boundary state, where {C = 0} is the minimum control target set (third step $\overline{){\rm{iii}}}$ in Fig. 1a).

Each of the three steps ($\overline{)\,{\rm{i}}\,}$, $\overline{)\,{\rm{ii}}\,}$ and $\overline{)\,{\rm{ii}}\,}$) can be summarized as in Fig. 1b: the first step requires b1 to b5 and the other steps requires b5 to b7. The first step begins to find a node (denoted by O) with no outgoing link (node G in b2 of Fig. 1b), which implies that O has no influence when identifying basin states in our framework. As a result, for each basin state *S* at the ℓ^th^ layer, there is a basin state at the ℓ^th^ layer which is equal to *S* except for the value of Ο. So, Ο is called a “symmetric node” of mean value 0.5 in the basin (Supplementary Figs 1–2). When identifying basin states, no influence of the symmetric node let us remove node O from the original network to repeat the process for a new symmetric node (node F in b2 of Fig. 1b) until there is no more symmetric node in the subnetwork. As in b2 and b3 in Fig. 1b, the original network is divided into two symmetric nodes (F and G) and a subnetwork without any symmetric node, respectively, where we refer to the subnetwork b3 as a “non-symmetric network”. The reduced attractor state of the non-symmetric network is the state obtained by removing all symmetric nodes from the desired attractor state, and the basin of the reduced attractor is referred to as “reduced basin”. Note that concatenating the values (0 or 1) of symmetric nodes and reduced basin states provides the exact basin. To reduce the computational time complexity when hierarchically calculating reduced basin states, we find sufficient conditions for terminal basin states. For instance, the B’s value in a given state *S* at the ℓ^th^ layer determines the unique value of *C*_ℓ+1_ in a solution candidate state at the (ℓ + 1)^th^ layer and the *C*_ℓ+1_’s unique value is an input to the E’s value in *S* at the ℓ^th^ layer (b3 of Fig. 1b), which implies that the B’s value in each basin state *S* must be an input to the E’s value in *S*. So, the algebraic structure among B, C and E gives an algebraic relation between two values of B and E in *S*. If *S* is not terminal, this relation must hold, where the condition in the contrapositive becomes a sufficient condition for *S* to be terminal (b4 of Fig. 1b and Supplementary Fig. 1). See “Sufficient conditions for terminal basin states and classifications of update rules” in the Methods section for details. By concatenating the values (0 or 1) of symmetric nodes (b2 of Fig. 1b) and the reduced basin states of the non-symmetric subnetwork (b5 of Fig. 1b), we can identify the basin of the desired attractor of the original network ($\overline{)\,{\rm{i}}\,}$ in Fig. 1b and Supplementary Fig. 2). Identification process of an exact basin is illustrated with an example network in Supplementary Fig. 3. The second and third steps begin to divide nodes of the non-symmetric network into three parts depending on the reduced basin (b5 in Fig. 1b): symmetric nodes of mean value 0.5, nodes of fixed values, and nodes of unfixed values in the reduced basin. For instance, in the case of the non-symmetric network b3, the reduced basin in Supplementary Fig. 1 gives no symmetric node, node C of a fixed value 1 (b6 in Fig. 1b) and the other nodes of no fixed values (b7 in Fig. 1b). So, every boundary state (A, B, C, D, E, F, G) from α (0, 0, 0, 0, 1, 0, 0) has C = 1 as well as (F, G) = (0, 0). Therefore comparing α and the states of (A, B, D, E) in each reduced basin state (Supplementary Fig. 1) provides mHD = 1, the boundary {(A, B, C, D, E, F, G) = (0, 0, 1, 0, 1, 0, 0)} and a minimum control set {C = 1} ($\overline{)\,{\rm{ii}}\,}$ and $\overline{){\rm{iii}}}$ in Fig. 1b and Supplementary Fig. 1).

### Application of BRC to a biomolecular network and analysis of the distribution of its average mHD

**Figure 2:**
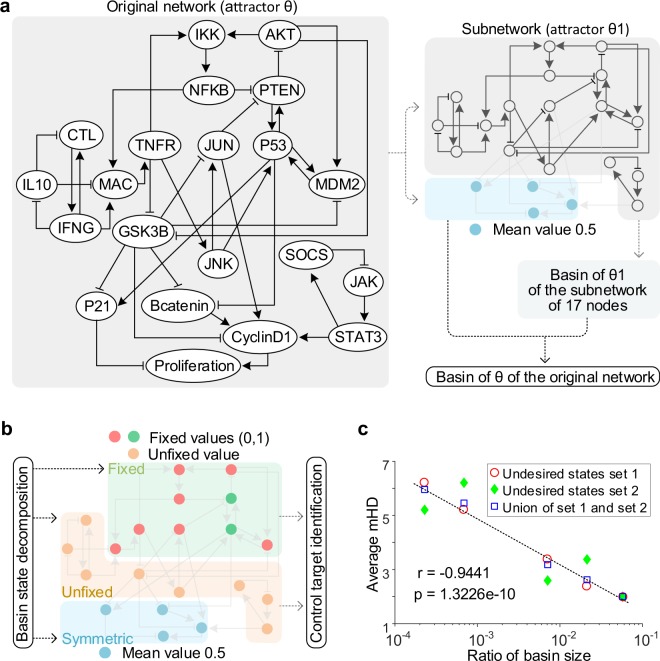
BRC of a biomolecular network and the distribution of its average mHD. (**a**) The overall process to identify all the desired basin states of a reduced colitis-associated colon cancer network of 21 nodes (referred to as “CACC21”). Arrows and bar-headed lines in CACC21 represent activation and inhibition, respectively. Symbol θ denotes a desired attractor with Proliferation of value 0, which is given in Supplementary Data 1 and Fig. 4. In upper right, CACC21 is divided into a subnetwork and four symmetric nodes of mean value 0.5 (cyan circles), where state θ1 of the subnetwork indicates the reduced attractor state of θ (Supplementary Fig. 4). The concatenation of the four symmetric nodes and each basin state of the subnetwork are used to identify the exact basin of θ (dotted arrow in bottom right), which is explained in Supplementary Fig. 4. (**b**) Basin state decomposition to identify control targets. Each basin state is decomposed into three sub-states of “Fixed” (cyan), “Unfixed” (orange) and “Symmetric” (red, green) nodes where “Symmetric” nodes are obtained from the structure of CACC21 and the other nodes are obtained from the reduced basin of θ1. Only “Fixed” and “Unfixed” nodes are used to identify control targets (two arrows on the right side), which is explained in Supplementary Fig. 5. (**c**) Decrease of average mHD with increase in basin size. CACC21 has two undesired attractors having the state value 1 for Proliferation (Supplementary Data 1). The undesired states sets 1 and 2 denote the sets of states converging to each of the undesired attractors. The letters “r” and “p” denote Pearson’s correlation coefficient and *P* value, respectively. See Supplementary Data 1 for details.

To show the details of BRC, we applied it to a biomolecular network of appropriate size, the reduced colitis-associated colon cancer (CACC) network of 21 nodes^14^ (Fig. 2a). This network is to be simply called “CACC21” and its state update logics are provided in Supplementary Data 1.

CACC21 is divided into the set of four symmetric nodes shown in the light blue area in Fig. 2a and the subnetwork of 17 non-symmetric nodes shown in the light gray area in Fig. 2a by using the CACC21 topology. The symmetric nodes of mean value 0.5 are identified hierarchically as shown in Fig. 1: Proliferation is the unique symmetric node in CACC21. P21 and CyclinD1 are symmetric in CACC21 without Proliferation. Bcatenin is symmetric in CACC21 without Proliferation, P21 and CyclinD1. There is no symmetric node in CACC21 without Proliferation, P21, CyclinD1 and Bcatenin. The subnetwork (top right of Fig. 2a) has a reduced attractor (θ1) composed of 17 non-symmetric nodes with their values in θ. As illustrated in Fig. 1, sufficient conditions for terminal basin states are derived to efficiently identify the basin of θ1 of the subnetwork, which will be then used to identify the basin of θ of the original network by concatenating the values of four symmetric nodes and each state of the basin of θ1 (Supplementary Fig. 4).

A basin state of θ can be decomposed as in in Fig. 2b: sub-states of symmetric nodes (“Symmetric”), nodes of fixed state values (“Fixed”) and the other nodes of unfixed values (“Unfixed”), where only the last two sub-states are used to identify control targets as in Fig. 1b (Supplementary Fig. 5).

CACC21 has two undesired attractors (state value 1 of Proliferation) and six desired attractors (state value 0 of Proliferation) in the state space. The average mHD from any state converging to one of the two undesired attractors to the basin boundary of one of the six desired attractors is presented in Fig. 2c. As expected, the average mHD decreases as the basin size of the desired attractor increases (Supplementary Data 1).

### BRC identifies therapeutic targets for temporary perturbation of a biomolecular network by identifying its exact basin

**Figure 3:**
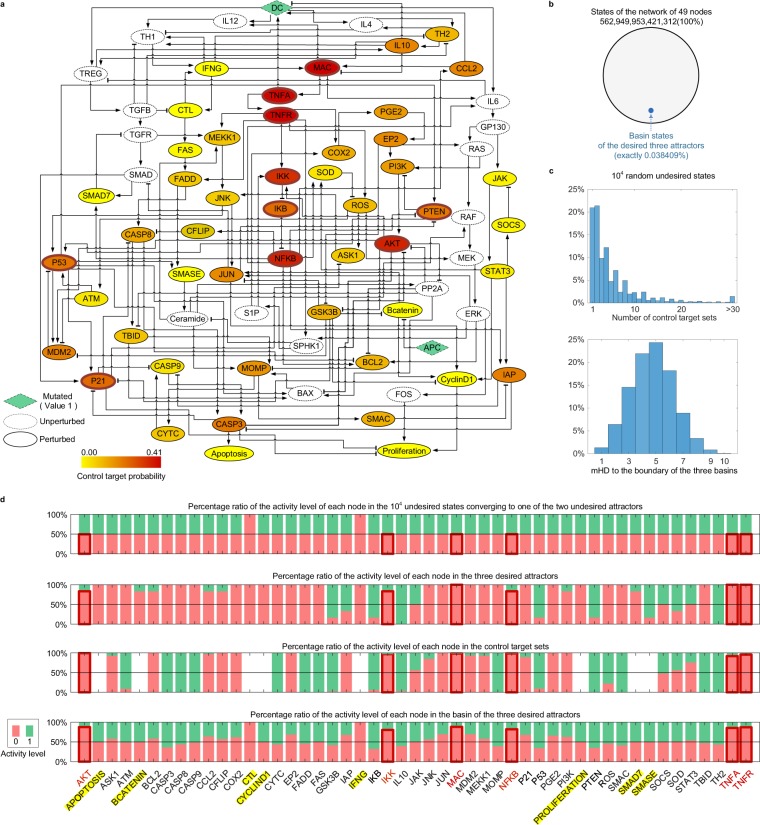
Application of BRC to the CACC49 network having a relatively small basin of the desired attractor. (**a**) CACC49 Boolean network with the probability of each node being a control target. Arrows and bar-headed lines represent activation and inhibition, respectively. Nodes DC and APC have mutated ON state (value 1), leading to fixed state values of the white nodes (dotted circles). Nodes with solid circles denote the candidate control targets. 10,000,000 randomly selected initial states are used to find out undesired attractors with constantly activated Proliferation (value 1) and desired attractors with constantly inactivated Proliferation (value 0), which are given in Supplementary Data 2. The darker red color of a node denotes a higher probability of being contained in control target sets given 10,000 randomly selected undesired states. (**b**) Sufficiently many basin states in relatively small size of a desired basin. Using the 10,000,000 randomly selected initial states, three desired attractors with constantly inactivated Proliferation are found (Supplementary Data 2). The size of desired basin states is exactly 0.038409% (21,622,344,760,959 states). (**c**) Distributions of the numbers of control target sets and mHD. Symbol “>30” in the top panel indicates that the number of control target sets is greater than 30. The average number of control target sets is 5.2476 (standard deviation 5.7703) and the average mHD is 4.7426 (standard deviation 1.5885). (**d**) Nodes enriched in control target sets. The activity levels range from 0 (inhibition) to 1 (activation). The first, second, third and last panels denote the activity level percentages of each node in 10,000 randomly selected undesired states, the desired attractors’ states, the control target sets and the desired basin states, respectively. The heights of boxes with red outlines in the four panels denote the activity level percentages of MAC, TNFA, TNFR, NFKB, AKT and IKK (darker red colors in **a**) being contained in control target sets, which are the top 6 control target nodes. The nodes marked with yellow backgrounds (APOPTOSIS, BCATENIN, CTL, CYCLIND1, IFNG, PROLIFERATION, SMAD7 and SMASE) indicate that they are not control target nodes (denoted by empties in the third panel).

CACC network originally consists of 70 nodes^14^. To represent the condition for a premalignant intestinal epithelial cell (IEC) in pro-tumor microenvironments, we fixed the values of DC and APC nodes to ON state^14^ (green diamonds in Fig. 3a). As a result, the states of 21 nodes are fixed and annotated as unperturbed nodes in Fig. 3a (dotted circles), and the remaining network consists of 49 nodes which are annotated as perturbed nodes in Fig. 3a (solid circles). We refer to the remaining subnetwork as “CACC49 network” or simply “CACC49” (Supplementary Data 2) which contains two phenotypic nodes, Apoptosis and Proliferation. We confirm that the dynamics of CACC49 can represent the growth of premalignant IECs^14^ (Supplementary Data 2) since the activation of Apoptosis was blocked and the activation of Proliferation was significantly increased whereas it did not reach its full activation.

From Monte-Carlo simulation, we found out 16 attractors (Supplementary Data 2). Two attractors have the constantly activated Proliferation node, which can be considered as undesired attractors (cancerous attractors) and so states converging to one of these two undesired attractors are considered undesired states (precancerous states). In addition, three attractors have the constantly inactivated Proliferation node (three desired attractors) and then states converging to one of these three desired attractors are considered desired states which are approximately 0.03994% of states in the state space. 10,000 randomly selected undesired states were used to identify boundary states.

Using our algorithm for identifying a basin, we found that desired states are 0.038409% out of all possible states. To drive the network state from an undesired state onto the boundary of the basin of a desired attractor by one-time temporary perturbation, we need to identify the boundary states that can be used to further find out control target sets and the mHD to the boundary, where the average number of control target sets is 5.2476 (standard deviation of 5.7703) and the average mHD is 4.7426 (standard deviation of 1.5885) as shown in Fig. 3c. Even if the relative basin size is extremely small as in Fig. 3b, the actual number of desired states is not much small (21,622,344,760,959 desired states), which might, in part, explain why the average mHD is not so large. The distribution of average mHD is theoretically and computationally obtained in Supplementary Figs 6–8, where we showed that the average and normalized average mHDs decrease as the network size increases but the relative size of a basin is fixed. In addition, we demonstrated that the mHD decreases if basin states are farther apart from each other in Supplementary Fig. 8.

From BRC point of view, each node in Fig. 3a is colored according to the relative frequency of inclusion of the node in control target sets, referred to as “control target probability” where a darker red color represents a higher control target probability. Activity levels of each node in the 10,000 randomly selected undesired states are uniformly distributed as in the first panel of Fig. 3d except for CTL and IFNG of the activity level 0 (first panel of Fig. 3d). The update rules for CTL and IFNG (CTL(t + 1) = IFNG(t) and IFNG(t + 1) = CTL(t)) and the fixed activity level 0 of CTL and IFNG in the three desired attractors (second panel of Fig. 3d and Supplementary Data 2) determine the fixed activity level of CTL and IFNG. So, CTL and IFNG become “fixed nodes”.

We found that eight nodes (i.e. APOPTOSIS, BCATENIN, CTL, CYCLIND1, IFNG, PROLIFERATION, SMAD7 and SMASE) are not included in control target nodes (third panel of Fig. 3d) since CTL and IFNG are fixed nodes and the other nodes are symmetric nodes of mean value of 0.5 (Fig. 3a and the fourth panel of Fig. 3d). We also found that six nodes (AKT, IKK, MAC, NFKB, TNFA and TNFR) are enriched in the control target sets (Fig. 3a and the third panel of Fig. 3d), where the inhibitory pattern is similar to that in the basin of the desired attractors (fourth panel of Fig. 3d). In addition, the six nodes showed lowest activities in the basin of the desired attractors in contrast to the undesired states (first panel of Fig. 3d) except the fixed nodes (CTL and IFNG). These results may indicate that the six nodes are essential for maintaining cancer progression, and so inhibition of them can result in cancer regression. The Akt signaling pathway is a well-known oncogenic pathway dysregulated in most colon cancer^15^ and inhibition of this pathway can prevent tumorigenesis of CAC^16^. IκB kinase (IKK) and Nuclear factor-κB (NF-κB) transcription factor are involved in the IKK/NF-κB signaling pathway linking inflammation and tumorigenesis of CAC^17,18^. TNF-α can augment the IKK/NF-κB signaling pathway, so blocking TNF-α and its receptor can reduce CAC^19^. Lastly, it remains as a future study to experimentally validate whether the activity of Mac1+ cells is critical to regulate CAC.

### BRC can reveal therapeutic targets for temporary perturbation of a biomolecular network without identifying its exact basin in the case of a relatively large basin

**Figure 4:**
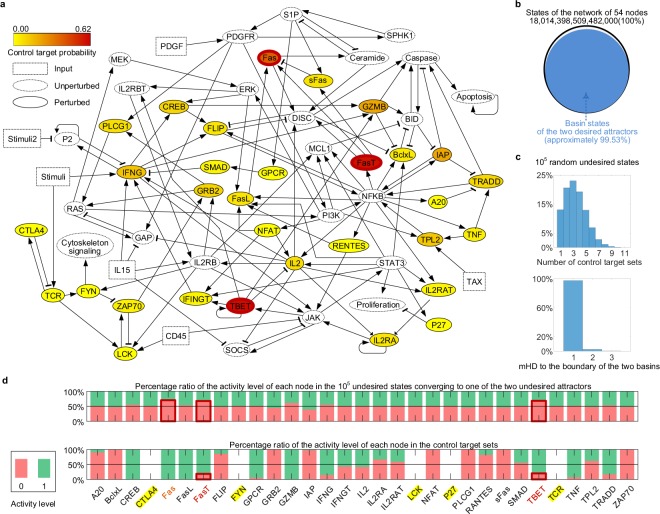
Application of BRC to the T-Cell54 network having a relatively large basin of the desired attractor. (**a**) T-Cell54 Boolean network with the probability of each node being a control target. Arrows and bar-headed lines represent activation and inhibition, respectively. Dotted rectangles denote input nodes where three inputs (Stimuli, IL15 and PDGF) are fixed to ON state and the remaining three inputs (CD45, Stimuli2 and TAX) are fixed to OFF. Dotted circles denote three outputs (Cytoskeleton signaling, Proliferation and Apoptosis), conceptual node P2 and known therapeutic target nodes for persistent intervention, which are not considered as candidates for temporary intervention targets. The remaining 31 nodes are marked with solid circles. 10,000,000 randomly selected initial states are used to find out undesired attractors (inactivated Apoptosis) and desired attractors (activated Apoptosis), which are given in Supplementary Data 3. The darker red color of a node indicates the higher probability of being included in control target sets given 100,000 randomly selected undesired states. (**b**) Sufficiently large size of the desired basin. Using the 10,000,000 randomly selected initial states, the total size of the basins of the desired attractors is approximately 99.53% (17,929,730,836,487,400 states). (**c**) Distributions of the numbers of control target sets and mHD. The average number of control targets is 3.3824 (standard deviation 1.6723). All the undesired states are situated at HD 3 at most and 97.35% of them have HD 1 to the desired basins. (**d**) Nodes enriched in control target sets. The activity levels range from 0 (inhibition) to 1 (activation). The top and bottom panels denote the activity level percentages of each node in the 100,000 randomly selected undesired states and the control target sets, respectively. The heights of boxes with red outlines in the two panels denote the activity level percentages of Fas, FasT and TBET (darker red colors in **a**) being contained in control target sets, which are the top 3 control target nodes. Nodes marked with yellow background (CTLA4, FYN, LCK, P27 and TCR) indicate non-target nodes (denoted by empties in the bottom panel).

In case the basin of a desired attractor is large enough such that it covers most of the state space, BRC can be applied without identifying the exact basin of the desired attractor. This is illustrated by a Boolean network model on survival of competent cytotoxic T lymphocytes in T cell large granular lymphocyte (T-LGL) leukemia^20,21^. This network consists of 60 nodes including proteins, mRNAs, and small molecules involved in T-LGL survival signaling pathways, together with six inputs (CD45, IL15, PDGF, Stimuli, Stimuli2 and TAX; dotted rectangles in Fig. 4a) and three outputs (Cytoskeleton signaling, Proliferation and Apoptosis). We refer to this model as “T-cell54” (Fig. 4a and Supplementary Data 3).

To reflect the condition for signaling abnormalities in T-LGL leukemia, we fixed the values of input nodes for Stimuli, IL15 and PDGF to ON state; and CD45, Stimuli2 and TAX to OFF state^21^. From Monte-Carlo simulation, we found out four attractors in which the values of two outputs Cytoskeleton signaling and Proliferation are 1 and 0, respectively. The other output Apoptosis has value 0 in two (“undesired attractors”) out of four attractors and value 1 in the other two attractors (“desired attractors”), where the total size of the basins of the desired attractors is approximately 99.53% (Fig. 4b and Supplementary Data 3).

Since the desired basins occupy most of the state space, every undesired state is expected to be located very close to the desired basins, which implies that perturbation of only few nodes at each undesired state might drive the undesired state onto the boundary of the desired basins. Except the inputs, the outputs, the conceptual node P2 and the previously identified 19 targets^20^ (dotted circles in Fig. 4a), the remaining 31 nodes (solid circles in Fig. 4a) were perturbed to examine whether such perturbation can actually drive the undesired state onto the boundary of a desired basin.

We identified boundary states from each of 100,000 random undesired states. As a result, we can find out control target sets and mHDs to the boundary of the two desired basins. The average number of control target sets is 3.3824 (standard deviation of 1.6723, upper panel of Fig. 4c). As expected, 10^5^ random undesired states are located very close to the boundary since 97.35% of all the undesired states are located with HD 1 to the boundary and 100% are situated at having HD 3 at most (lower panel of Fig. 4c).

Each node in Fig. 4a is colored according to the relative frequency of its inclusion in all control target sets where a darker red color represents a higher probability of being a control target. TBET, Fas and FasT are highly enriched in the control target sets (Fig. 4a), where their activity levels are opposite to those in the 100,000 random undesired states (Fig. 4d). Fas and FasT are involved in the signaling pathway for Fas-induced apoptosis^22^, and they were also previously suggested as potential therapeutic targets from the analysis of the ODE model of T-LGL network^23^. We further found that TBET (T box expressed in T cells) can be a candidate for a therapeutic target.

### Comparison of temporary vs. persistent interventions in controlling a biomolecular network

**Figure 5:**
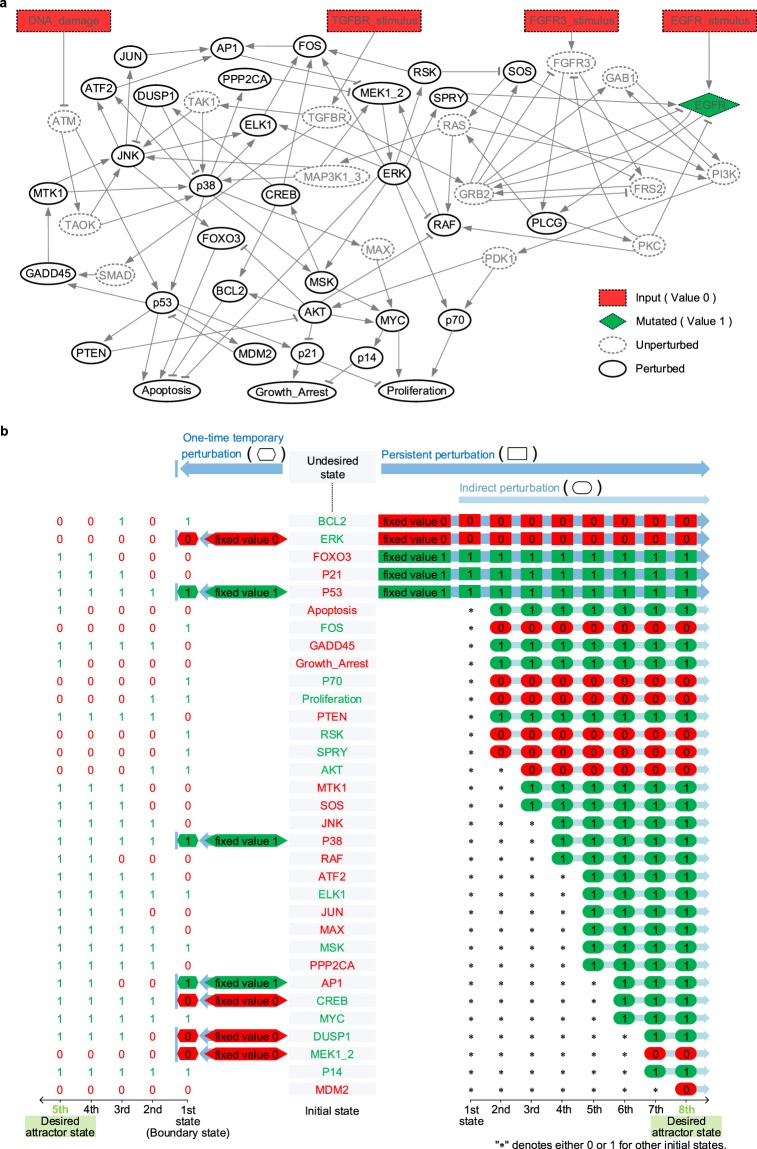
Temporary vs. persistent interventions. (**a**) MAPK network for urinary bladder cancer with EGFR over-expression. Arrows and bar-headed lines represent activation and inhibition, respectively. Dotted rectangles denote input nodes of value 0 and a diamond-shaped square denotes over-expressed EGFR of value 1. The 20 nodes marked with dotted circles (“unperturbed”) have fixed values. The remaining 33 nodes (“perturbed”) are marked with solid circles (Supplementary Data 4). (**b**) Comparison of temporary vs. persistent interventions. The green and red colors of the 33 nodes in the center denote their undesired state values 1 and 0, respectively. The 5^th^ and 8^th^ columns on the left and right sides denote the desired attractor state, respectively. The initial and final of arrows (blue) denote the start and end of perturbations, respectively, where bars on the left side denote the end of temporary perturbation. On the left side, the 1^st^ state denotes a boundary state, where hexagons denote temporary target nodes and their values. We obtained the 2^nd^, 3^rd^, 4^th^ and 5^th^ states by substituting the boundary state into the update rules (Supplementary Data 4) as an initial state, where the 5^th^ state denotes the desired attractor state. On the right side, the 1^st^ state denotes any state such that the persistent target nodes’ values are fixed (rectangles in red or green) and the other nodes’ values can be 0 or 1 (marked with*). Substituting any state in the 1^st^ state into the update rules as an initial state, 9 non-persistent target nodes have fixed values at the right next time step, which are marked with circles in red or green at the 2^nd^ state. Similarly, substituting any state in the 2^nd^ state into the update rules as an initial state, 12 non-persistent target nodes have fixed values at the right next time step, which are marked with circles in red or green at the 3^rd^ state. Repeating this process, all nodes have fixed values by substituting any state in the 7^th^ state into the update rules as an initial state.

To compare the differential effects of temporary and persistent perturbations^7–11^, we employed the Boolean network model of mitogen-activated protein kinase (MAPK) network^24^ composed of 53 nodes (Fig. 5a and Supplementary Data 4). We fixed the values of EGFR to ON (dotted diamond in Fig. 5a) and all the input nodes to OFF (dotted rectangles in Fig. 5a) for the case of urinary bladder cancer with EGFR over-expression^24^. As a result, the state values of 20 nodes are also fixed, which are annotated as unperturbed nodes (dotted circles in Fig. 5a), and the remaining network consists of 33 nodes which are annotated as perturbed nodes (solid circles in Fig. 5a). We refer to this subnetwork as “MAPK33” (update rules in Supplementary Data 4).

10,000,000 randomly selected initial states converge to one of 12 attractors (Supplementary Data 4). Among those, one desired attractor is an attractor with (Apoptosis, Growth_Arrest, Proliferation) = (1, 1, 0), which is located in the 5th and 8th columns of Fig. 5b, and one undesired attractor is an attractor with (Apoptosis, Growth_Arrest, Proliferation) = (0, 0, 1). An undesired state is the undesired attractor with the values denoted by the color of each node (red and green denote values 0 and 1, respectively), which is located in the center of Fig. 5b. A temporary target set for BRC is (ERK, P53, P38, AP1, CREB, DUSP1, MEK1_2) = (0, 1, 1, 1, 0, 0, 0) (hexagons on Fig. 5b, left) and a persistent target set for persistent perturbation is (BCL2, ERK, FOXO3, P21, P53) = (0, 0, 1, 1, 1) (rectangles in Fig. 5b, right).

After one-time temporary perturbation of the temporary target set, the undesired state is driven to the boundary state (“1st state” on the left side of Fig. 5b), which converges to the desired attractor without any further perturbation. When applying BRC, there is no indirect perturbation of a node. On the other hand, if we persistently perturb the persistent target nodes, as usually assumed in most previous studies on complex network control, then the other nodes can also be indirectly perturbed and any state converges to the desired attractor through 8 state transitions in our Boolean simulation (right of Fig. 5b and Methods). In addition, the conventional control methods could not guarantee to achieve the control goal, convergence to the desired attractor, if the persistent perturbation is interrupted before an initial state converges to the desired attractor. From the simulation results with different interruptions during perturbation simulations (Supplementary Fig. 9a), we found that the success rate of converging to the desired attractor from random initial states exponentially decreases as the interruption occurs at an earlier state transition step. Hence, insufficient duration of persistent perturbation may fail to achieve the desired control goal. Intriguingly, in this example, none of the states in the undesired basin could successfully be controlled over such insufficient perturbation duration (Supplementary Fig. 9b).

### A determining factor for the control target nodes and their perturbation values

**Figure 6:**
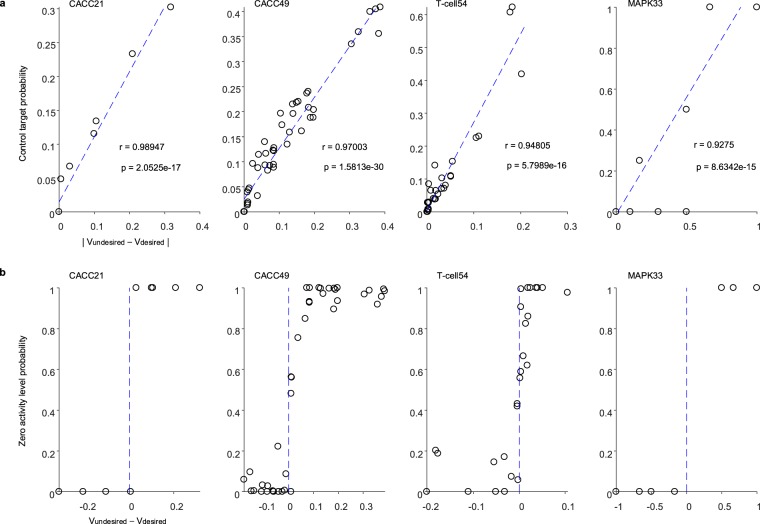
Relation of control target and activity level probabilities with the difference of undesired and desired states. (**a**) Correlation analysis between control target probability and |V_undesired_ − V_desired_| of each node in CACC21, CACC49, T-cell49 and MAPK33 networks (Supplementary Data 1, 2, 3 and 4). Symbol V_undesired_ (V_desired_) denotes the mean value of a node state in all undesired (desired) states, where the undesired and desired states in CACC21, CACC49, T-cell49 and MAPK33 are defined in the previous subsections. |V_undesired_ − V_desired_| denotes the absolute value of difference between V_undesired_ and V_desired_ of each node. Pearson’s correlation coefficients (r) and *P* values (p) indicate a strong positive correlation. (**b**) Relation between zero activity level probability and V_undesired_ − V_desired_ of each node in CACC21, CACC49, T-cell49 and MAPK33 networks (Supplementary Data 1, 2, 3 and 4). The zero activity level probability of a node denotes the probability that the node’s value is 0 in all the control target sets, which tends to increase as V_undesired_ − V_desired_ increases.

Identifying control target nodes depends on both a desired basin and undesired states. A question then arises as to whether there is any particular dependence relationship between these two factors. To answer to this question, we analyzed all the nodal values in both undesired and desired basins and found that the difference between nodal values in the two basins may provide a clue. We discovered that a larger difference (x-axis in Fig. 6a) tends to result in a higher probability of the node to be a control target (y-axis in Fig. 6a). Moreover, the sign of the difference (x-axis in Fig. 6b) can be used to determine inhibition or activation of the control target (y-axis in Fig. 6b). Together, these results suggest that control targets and their mode (i.e. activation or inhibition) of control in BRC can be predicted from the information of undesired and desired states without computing the distance from an undesired state to a desired basin.

## Discussion

Systems biological investigations primarily focused on unraveling the hidden mechanism of biological phenomena^25–38^ and they are recently being extended to controlling the dynamic behavior caused by complex regulatory networks. Previous studies of controlling complex networks mostly considered persistent perturbation of control target elements in the networks. Such persistent perturbation might, however, not be feasible in some cases or bring about undesired side effects even if it is feasible. To resolve this issue fundamentally, we proposed in this study a different concept of network control, BRC based on temporary perturbation. The basic idea of this control strategy is to drive any undesired state to a boundary state of the basin of attraction of a desired attractor using temporary perturbation, whereafter the network state converges to the desired attractor without any further intervention. To implement BRC, we need to find out the exact basin of attraction to a given desired attractor and the boundary of the basin. We have developed general algorithms for these purposes. In case where the size of basin is large enough, BRC can be implemented without identifying the exact basin as illustrated in the example of Fig. 4.

Of note BRC can also be used to the case of a desired cyclic attractor by temporarily perturbing the identified control target nodes to some fixed state values whereas previous network control strategies using persistent perturbation require periodically changing the state values of control target nodes^8,10^, which is very difficult or mostly impossible in many cases. For instance, oscillations emerge in various cellular functions^39,40^ such as cell cycle, DNA damage response with p53, and stress response with NF-kB, and they are all represented by cyclic attractors of the underlying molecular networks^41,42^. In those cases, periodically changing any target molecule is very hard and not feasible in practice. On the other hand, BRC requires only to temporarily perturb some target nodes to fixed state values such that the network state transits to the boundary of the basin of attraction to the desired cyclic attractor. There is another important feature of BRC. Owing to the nature of its transient perturbation, BRC preserves the original attractor landscape of a complex network whereas it is often distorted by persistent perturbation^7^. This means that the desired cyclic attractor might change or even disappear unexpectedly if persistent perturbation is applied to the network, but such cases would not happen if we employ BRC. In the case of p53 regulation, the cellular phenotype of an oscillatory p53 response can be conversely changed to that of a sustained p53 response^43^ and therefore preserving the original attractor landscape is critical to induce a desired phenotype.

We have provided examples of an ecological network^44^ and an insect social network^45^ to show the applicability of BRC to diverse complex networks. In the ecological network, the ecological community assembly consists of three plants species and two pollinators species, and a control target set was found to restore an ecologically desired steady state from an undesired state^44^ (Supplementary Fig. 10). The “control target nodes” found from BRC indicate key structural components (or “master regulators”) of information flow in the insect social network composed of paired dominant interactions in the social wasp *R. marginata* where physical interactions play an important role in information flow and a primitively eusocial wasp is dominating over the other in paired interactions^45^ (Supplementary Fig. 11). A dominant in a paired interaction usually becomes a subordinate in other paired interaction. Since the dominant network consists of a feedforward structure without inhibition of information flow, there exist dominant wasps which are not subordinates; and such wasps play roles as “master regulators” with respect to information flow. We showed that the set of master regulators are equal to the set of control target nodes found from BRC without constructing the hierarchical structure of the paired interactions (Supplementary Fig. 11).

The number of control targets is critical for application to real systems, and it depends on the size and structure of the basin of a desired attractor. This number becomes smaller for a larger basin (Fig. 2c). On the other hand, if the size of basin is relatively small compared to the size of a network, one may intuitively imagine that a larger number of control targets would be needed. However, it is not as shown in Fig. 3 where the average number of control targets is about 5 (note that the Hamming distance to the basin is 5) which is very small considering that the network consists of 49 nodes (so 2^49^ states in total) and the relative size of the basin is extremely small (about 0.0384% of 2^49^). Even if the relative size of basin is extremely small in this case, the number of basin states is sufficiently large (above 10^11^) enough to cover all 10^4^ random undesired states within average HD of 5, which might in part explain the counter-intuitive result. To reduce the number of control targets, a union of basins of all desired attractors, instead of considering only one desired attractor, can be used for BRC (Supplementary Fig. 8c). In this study, we did not consider any preprocessing procedure to reduce the number of control targets before applying BRC, and therefore it remains as a further study.

Our deterministic control framework cannot reflect fluctuations in biological systems when identifying temporary control target sets. Even if an undesired state is driven to a boundary state converging to a desired attractor by using the temporary control targets, the boundary state could switch to an undesired state due to the fluctuation. If the fluctuation is considered a random process of flipping the state of each node, determining a control target set for a boundary state stable to the flipping might be a way to reflect the fluctuation (Supplementary Fig. 12). To reflect the fluctuation in the process of identifying control targets, a stochastic control framework should be considered^46^.

Our discrete control framework might be extended to non-discrete models including ordinary differential equation (ODE) models if the exact basin of a given attractor could be explicitly identified. We demonstrated the possibility by using an example of a homogeneous system of finite linear differential equations with constant coefficients in Supplementary text. In the case that a non-linear ODE model and a desired point attractor state are given, the non-linear ODE can be linearized at the desired state and the linearized ODE might provide desired basin states around the desired state. Such a linearization might be a starting point to find an approximate basin of the desired attractor.

So far, there has been no study considering minimal one-time temporary intervention for complex network control and no attempt to identify the exact basin of attraction to a desired attractor using the topological and algebraic structures of a network. Hence, the presented control strategy and algorithms for identifying the exact basin of attraction would be useful for any future studies of controlling complex networks with minimal interventions.

## Methods

### Classifications of nodes

We consider a Boolean network *G*(*V*_0_, *E*_0_, *F*_0_) with synchronous update rules for its state transition where *V*_0_, *E*_0_ and *F*_0_ are the sets of nodes, directed edges between nodes, and update rules for the nodes, respectively. Node *x* is called an “input to *y*” if there exists an outgoing link from *x* to *y*. The outdegree and indegree of *x* are the numbers of outgoing links from *x* and incoming links to *x*, respectively, which are denoted by *outdeg*(*x*) and *indeg*(*x*). *x* is also used to denote the state value of node *x*. If *outdeg*(*x*) = 0, then *x* is called an “independent” node. Let *G*(*V*_1_, *E*_1_, *F*_1_) denote the subnetwork obtained by removing all independent nodes from *G*(*V*_0_, *E*_0_, *F*_0_). If *G*(*V*_1_, *E*_1_, *F*_1_) has an independent node, then we remove all independent nodes from *G*(*V*_1_, *E*_1_, *F*_1_). We repeat this removal process until there is no more independent node in subnetwork *G*(*V*_*η*_, *E*_*η*_, *F*_*η*_) for some positive integer *η*. Independent nodes in *G*(*V*_*i*_, *E*_*i*_, *F*_*i*_) (0 ≤ *i* ≤ *η* − 1) are also called “symmetric” nodes since independent nodes generate a symmetric structure in the basin of each attractor: Let ${V}_{0}=\{{x}_{1},\cdots ,{x}_{{n}_{0}}\}$ and $\{{x}_{j}^{sym}\in {V}_{0}|1\le j\le {n}_{sym}\}$ be the set of symmetric nodes in *G*(*V*_*i*_, *E*_*i*_, *F*_*i*_)(0 ≤ *i* ≤ *η* − 1) for some positive integers *n*_0_ and *n*_*sym*_. If $({x}_{1},\cdots ,{x}_{j}^{sym},\cdots ,{x}_{{n}_{0}})$ is a basin state of an attractor, then $({x}_{1},\cdots ,1-{x}_{j}^{sym},\cdots ,{x}_{{n}_{0}})$ is also a basin state of the attractor since an independent node does not regulate any node, which is explained in “Sufficient conditions for terminal basin states and classifications of update rules” (see below). Both $({x}_{1},\cdots ,{x}_{j}^{sym},\cdots ,{x}_{{n}_{0}})$ and $({x}_{1},\cdots ,1-{x}_{j}^{sym},\cdots ,{x}_{{n}_{0}})$ are basin states, and so this is the reason why independent nodes are called symmetric nodes, which results in average value 0.5 of every symmetric node in the basin. Node *x* in *G*(*V*_*η*_, *E*_*η*_, *F*_*η*_) is called “deterministic” if there exists a node of indegree 1 to which *x* is an input. Node *x* in *G*(*V*_*η*_, *E*_*η*_, *F*_*η*_) is called “nondeterministic” if *x* is not deterministic. Let ${V}_{det}=\{{x}_{\ell }^{det}\in {V}_{\eta }|1\le \ell \le {n}_{det}\}$ and ${V}_{nondet}=\{{x}_{\theta }^{nondet}\in {V}_{\eta }-{V}_{det}|1\le \theta \le {n}_{nondet}\}$ be the sets of deterministic and nondeterministic nodes, respectively. Let ${V}_{nondet}^{inputdet}=\{{x}_{\tau ,inputdet}^{nondet}\in {V}_{nondet}|1\le \tau \le {n}_{nondet}^{inputdet}\}$ be the set of nondeterministic nodes which have at least one deterministic node as an input.

### Sufficient conditions for terminal basin states and classifications of update rules

The basin states of an attractor are hierarchically identified by following the reverse of state transition trajectories, where the attractor is located at the 1^st^ layer in the basin. Basin states calculated from a basin state at the δ^th^ layer are located at the (δ + 1)^th^ layer. Let *x*(*t* + 1) and *x*(*t*) denote state values of node *x* at time steps *t* + 1 and *t*, respectively, which are located in the δ^th^ and (δ + 1)^th^ layers (δ ≥ 1), respectively. For each deterministic node ${x}_{\ell }^{det}$, let ${x}_{{\ell }_{j}}^{det}(1\le j\le {n}_{det,\ell })$ be nodes of indegree 1 to which ${x}_{\ell }^{det}$ is an input. The update rule ${x}_{{\ell }_{1}}^{det}(t+1)={f}_{det,{\ell }_{1}}({x}_{\ell }^{det}(t))$ is called a “deterministic equation” since the equation determines the unique value of ${x}_{\ell }^{det}$

1 $${x}_{\ell }^{det}(t)={f}_{det,{\ell }_{1}}^{-1}({x}_{{\ell }_{1}}^{det}(t+1))$$

given the value of ${x}_{{\ell }_{1}}^{det}(t+1)$, where the other update rules

$${x}_{{\ell }_{j}}^{det}(t+1)={f}_{det,{\ell }_{j}}({x}_{\ell }^{det}(t))\,({\rm{2}}\le j\le {n}_{det,\ell })$$

are used as necessary conditions for given states at time step *t* + 1 to have basin states at time step *t* as follows:

$${x}_{\ell }^{det}(t)={f}_{det,{\ell }_{1}}^{-1}({x}_{{\ell }_{1}}^{det}(t+1))={f}_{det,{\ell }_{j}}^{-1}({x}_{{\ell }_{j}}^{det}(t+1))\,(2\le j\le {n}_{det,\ell }).$$

Then the contrapositive of the conditional statements gives ${n}_{det,\ell }-1$ sufficient conditions for given states at time step *t* + 1 to be terminal basin states as follows:

2 $${f}_{det,{\ell }_{1}}^{-1}({x}_{{\ell }_{1}}^{det}(t+1))=1-{f}_{det,{\ell }_{j}}^{-1}({x}_{{\ell }_{j}}^{det}(t+1))\,(2\le j\le {n}_{det,\ell })$$

A different type of sufficient conditions for terminal basin states can be constructed from the update rule for ${x}_{\tau ,inputdet}^{nondet}$, a nondeterministic node which has at least one deterministic node as an input,

$${x}_{\tau ,inputdet}^{nondet}(t+1)={f}_{\tau ,inputdet}^{nondet}({x}_{{\ell }^{\tau ,1}}^{det}(t),\cdots ,{x}_{{\ell }^{\tau ,i}}^{det}(t),{x}_{{\theta }^{\tau ,1}}^{nondet}(t),\cdots ,{x}_{{\theta }^{\tau ,s}}^{nondet}(t)),$$

where ${x}_{{\ell }^{\tau ,m}}^{det}\in {V}_{det}(1\le m\le i)$ and ${x}_{{\theta }^{\tau ,m}}^{nondet}\in {V}_{indet}(1\le m\le s)$. Let us consider basin states at time *t* + 1 which do not satisfy (Eq. 2). Then, the equality

3 $${x}_{\tau ,inputdet}^{nondet}(t+1)=1-{f}_{\tau ,inputdet}^{nondet}({f}_{det,{\ell }_{1}^{\tau ,1}}^{-1}({x}_{{\ell }_{1}^{\tau ,1}}^{det}(t+1)),\cdots ,{f}_{det,{\ell }_{1}^{\tau ,i}}^{-1}({x}_{{\ell }_{1}^{\tau ,i}}^{det}(t+1)),\ast ,\cdots ,\ast )$$

becomes also sufficient conditions for given states at time step *t* + 1 to be terminal basin states, where “*” denotes either 0 or 1. Therefore, (Eq. 2) and (Eq. 3) are sufficient conditions for given states at time step *t* + 1 to be terminal basin states. The update rules are called “nondeterministic equations” except for “deterministic equation”.

## Algorithm for identifying the exact basin of a desired attractor

Step 1. Determine a desired attractor. Let us consider one attractor of interest (e.g. a desired attractor) in the state space of a Boolean network *G*(*V*_0_, *E*_0_, *F*_0_). Let *β* = [*S*_1_, ⋯, *S*_*c*_] be a desired attractor of length *c*(*c* ≥ 1), where *S*_*q*_(1 ≤ *q* ≤ *c*) is a state in the state space; substitution of *S*_*p*_ into the update rule as an initial state results in the state *S*_*p*+1_(1 ≤ *p* ≤ *c* − 1) at the next time step; substitution of *S*_*c*_ into the update rule as an initial state results in *S*_1_ at the next time step.

Step 2. Classify nodes in the network. Nodes are classified into three categories: “symmetric”, “deterministic” and “nondeterministic” nodes by following the definitions in the foregoing subsection (Classifications of nodes).

Step 3. Determine the subnetwork without symmetric nodes. Following the removing process of symmetric nodes in the foregoing subsection (Classifications of nodes), the desired subnetwork *G*(*V*_*η*_, *E*_*η*_, *F*_*η*_) can be obtained.

Step 4. Identify the basin of the reduced desired attractor of the subnetwork without symmetric nodes. Let $\tilde{\beta }=[{\tilde{S}}_{1},\cdots ,{\tilde{S}}_{c}]$ be the reduced attractor in the state space of *G*(*V*_*η*_, *E*_*η*_, *F*_*η*_), where ${\tilde{S}}_{i}$ is obtained from *S*_*i*_ (1 ≤ *i* ≤ *c*) by removing the values of nodes that are not contained in *V*_*η*_.

Step 4-1. Classify Boolean update rules for nodes in *G*(*V*_*η*_, *E*_*η*_, *F*_*η*_). The update rules for nodes in *G*(*V*_*η*_, *E*_*η*_, *F*_*η*_) are classified into three categories: “deterministic equations”, “sufficient conditions for terminal basin states” and “nondeterministic equations” by following the definitions in the foregoing subsection (Sufficient conditions for terminal basin states and classifications of update rules).

Step 4-2. Construct the collection Ω_*ternimal*_ of all terminal basin states converging to $\tilde{\beta }$. Using (Eq. 2) and (Eq. 3), we can identify the collection of all the terminal basin states.

Step 4-3. Identify all states converging to each ${\tilde{S}}_{q}(1\le q\le c)$, the set of which is called the “local basin of ${\tilde{S}}_{q}$” and denoted by $LB({\tilde{S}}_{q})$. Since local basins have no common state except for ${\tilde{S}}_{q}(1\le q\le c)$, we can find each $LB({\tilde{S}}_{q})$ in hierarchical and parallel processing where the first layer consists of ${\tilde{S}}_{q}$.

### Case 1.

If ${\tilde{S}}_{q}$ is contained in Ω_*ternimal*_, then $LB({\tilde{S}}_{q})=\{{\tilde{S}}_{q}\}$.

Otherwise, go to Case 2.

### Case 2.

Let ${\tilde{S}}_{q}=({v}_{1}^{q},\cdots ,{v}_{n}^{q})$ with ${v}_{i}^{q}\in {V}_{det}$ and ${v}_{j}^{q}\in {V}_{nondet}$,

where *n* = *n*_*det*_ + *n*_*nondet*_, 1 ≤ *i* ≤ *n*_*det*_ and *n*_*det*_ + 1 ≤ *j* ≤ *n*,

where *V*_*det*_ and *V*_*nondet*_ are defined in the foregoing subsection (Classifications of nodes). The second layer consists of solutions

$$({x}_{1}^{det},\cdots ,{x}_{{n}_{det}}^{det},{x}_{1}^{nondet},\cdots ,{x}_{{n}_{nondet}}^{nondet})=({v}_{1}^{det},\cdots ,{v}_{{n}_{det}}^{det},{v}_{1}^{nondet},\cdots ,{v}_{{n}_{nondet}}^{nondet})$$

of the system of Boolean equations

$${v}_{\theta }^{q}={f}_{\theta }({x}_{1}^{det},\cdots ,{x}_{{n}_{det}}^{det},{x}_{1}^{nondet},\cdots ,{x}_{{n}_{nondet}}^{nondet})\,\,({n}_{det}+1\le \theta \le n),$$

where $({x}_{1}^{det},\cdots ,{x}_{{n}_{det}}^{det})=({v}_{1}^{det},\cdots ,{v}_{{n}_{det}}^{det})$ is determined by (Eq. 1) with ${x}_{{\ell }_{i}}^{det}(t+1)={v}_{i}^{q}(1\le i\le {n}_{det})$. Go to Case 3.

### Case 3.

If there is a basin state in the second layer, then replace ${\tilde{S}}_{q}$ with each state in the second layer and repeat Cases 1 and 2, so that the third layer can be obtained. Repeat this process until all the given states are contained in Ω_*ternimal*_. Then we can finally identify $LB({\tilde{S}}_{q})(1\le q\le c)$.

Step 4-4. Identify the basin of $\tilde{\beta }=[{\tilde{S}}_{1},\cdots ,{\tilde{S}}_{c}]$ in the subnetwork *G*(*V*_*η*_, *E*_*η*_, *F*_*η*_). The union of local basins $\mathop{\cup }\limits_{1\le q\le c}LB({\tilde{S}}_{q})$ is the basin of the reduced attractor $\tilde{\beta }$.

Step 5. Identify the exact basin of *β* = [*S*_1_, L, *S*_*c*_] in *G*(*V*_0_, *E*_0_, *F*_0_). Let $\{{x}_{j}^{sym}\in {V}_{0}|1\le j\le {n}_{sym}\}$ be the set of symmetric nodes. Then each state of the exact basin can be represented as

$$({x}_{1}^{sym},\cdots ,{x}_{{n}_{sym}}^{sym},{x}_{1}^{det},\cdots ,{x}_{{n}_{det}}^{det},{x}_{1}^{nondet},\cdots ,{x}_{{n}_{nondet}}^{nondet})=({v}_{1}^{sym},\cdots ,{v}_{{n}_{sym}}^{sym},{v}_{1}^{det},\cdots ,{v}_{{n}_{det}}^{det},{v}_{1}^{nondet},\cdots ,{v}_{{n}_{nondet}}^{nondet}),$$

where $${v}_{j}^{sym}\in \{0,1\}(1\le j\le {n}_{sym})$$ and $$({v}_{1}^{det},\cdots ,{v}_{{n}_{det}}^{det},{v}_{1}^{nondet},\cdots ,{v}_{{n}_{nondet}}^{nondet})\in \mathop{\cup }\limits_{1\le q\le c}LB({\tilde{S}}_{q})$$.

## Identification of control targets for BRC

Let *β* = [*S*_1_, ⋯, *S*_*c*_] be the attractor in the foregoing subsection (Algorithm for identifying the exact basin of a desired attractor). Let *α* be an undesired state converging to an undesired attractor. Let $\mathop{\cup }\limits_{1\le q\le c}LB({\tilde{S}}_{q})$ be the basin of the reduced attractor $\tilde{\beta }$ in the state space of *G*(*V*_*η*_, *E*_*η*_, *F*_*η*_), which are defined in the foregoing subsections.

Step 1. Classify nodes in *G*(*V*_*η*_, *E*_*η*_, *F*_*η*_) depending on state values in $$\mathop{\cup }\limits_{1\le q\le c}LB({\tilde{S}}_{q})$$. Let $${V}_{sym}=$$$$\{{x}_{j}^{sym}\in {V}_{\eta }|1\le j\le {n}_{sym}\}$$ and $${V}_{fixed}=\{{x}_{j}^{fixed}\in {V}_{\eta }-{V}_{sym}|1\le j\le {n}_{fixed}\}$$ be the sets of symmetric nodes $${x}_{i}^{sym}$$ and nodes $${x}_{j}^{fixed}$$ of fixed values in $$\mathop{\cup }\limits_{1\le q\le c}LB({\tilde{S}}_{q})$$, respectively, where the fixed values are denoted by $${x}_{j}^{fixed}={v}_{j}^{fixed}\in \{0,1\}$$. Let the set of nodes of unfixed values in $$\mathop{\cup }\limits_{1\le q\le c}LB({\tilde{S}}_{q})$$ be denoted by $${V}_{unfixed}=\{{x}_{j}^{unfixed}\in {V}_{\eta }-$$$${V}_{sym}-{V}_{fixed}|1\le j\le {n}_{unfixed}\}$$. Here, “symmetric” means that if $$({x}_{1}^{sym},\cdots ,{x}_{{n}_{sym}}^{sym},{v}_{1}^{fixed},\cdots ,{v}_{{n}_{fixed}}^{fixed},{x}_{1}^{unfixed},\cdots ,$$$${x}_{{n}_{unfixed}}^{unfixed})\in \mathop{\cup }\limits_{1\le q\le c}LB({\tilde{S}}_{q})$$, then

$$(1-{x}_{1}^{sym},\cdots ,1-{x}_{{n}_{sym}}^{sym},{v}_{1}^{fixed},\cdots ,{v}_{{n}_{fixed}}^{fixed},{x}_{1}^{unfixed},\cdots ,{x}_{{n}_{unfixed}}^{unfixed})\in \mathop{\cup }\limits_{1\le q\le c}LB({\tilde{S}}_{q}).$$

Step 2. Represent *α* depending on the classifications. Using the notations defined in Step 1, the undesired state *α* can be represented as

$$\alpha =({\alpha }_{1}^{sym},\cdots ,{\alpha }_{{n}_{sym}}^{sym},{\alpha }_{1}^{fixed},\cdots ,{\alpha }_{{n}_{fixed}}^{fixed},{\alpha }_{1}^{unfixed},\cdots ,{\alpha }_{{n}_{unfixed}}^{unfixed}).$$

Step 3. Calculate the minimum Hamming distance (mHD) from *α* to $\mathop{\cup }\limits_{1\le q\le c}LB({\tilde{S}}_{q})$.

Let $HD(({\alpha }_{1}^{fixed},\cdots ,{\alpha }_{{n}_{fixed}}^{fixed}),({v}_{1}^{fixed},\cdots ,{v}_{{n}_{fixed}}^{fixed}))={\rho }_{fixed}$ be the Hamming distance between the two states $({\alpha }_{1}^{fixed},\cdots ,{\alpha }_{{n}_{fixed}}^{fixed})$ and $({v}_{1}^{fixed},\cdots ,{v}_{{n}_{fixed}}^{fixed})$ such that ${\alpha }_{{p}_{i}}^{fixed}=1-{v}_{{p}_{i}}^{fixed}$ for some *p*_*i*_ ∈ {1, ⋯, *n*_*fixed*_}(1 ≤ *i* ≤ *ρ*_*fixed*_). Let $HD(({\alpha }_{1}^{unfixed},\cdots ,{\alpha }_{{n}_{unfixed}}^{unfixed}),({v}_{1}^{unfixed},\cdots ,{v}_{{n}_{unfixed}}^{unfixed}))={\rho }_{unfixed}$ be the minimum Hamming distance between two vectors of nodes of unfixed values in *α* and $\mathop{\cup }\limits_{1\le q\le c}LB({\tilde{S}}_{q})$ such that

$${\alpha }_{{q}_{1}}^{unfixed}=1-{v}_{{q}_{1}}^{unfixed},\cdots ,{\alpha }_{{q}_{{\rho }_{unfixed}}}^{unfixed}=1-{v}_{{q}_{{\rho }_{unfixed}}}^{unfixed}\,\mathrm{for}\,({q}_{1},\cdots ,{q}_{{\rho }_{unfixed}})\in I,$$

where *I* is an index set contained in {1, ⋯, *n*_*unfixed*_}. Therefore, mHD between *α* and $\mathop{\cup }\limits_{1\le q\le c}LB({\tilde{S}}_{q})$ is *ρ*_*fixed*_ + *ρ*_*unfixed*_.

Step 4. Identify control target sets driving *α* to the boundary of the exact basin. Let |*I*| denote the number of elements of *I*. Then there exist |*I*| control target sets

$$\{{x}_{{p}_{1}}^{fixed},\cdots ,{x}_{{p}_{{\rho }_{fixed}}}^{fixed},{x}_{{q}_{1}}^{unfixed},\cdots ,{x}_{{q}_{{\rho }_{unfixed}}}^{unfixed}|\begin{array}{c}{x}_{{p}_{i}}^{fixed}=1-{\alpha }_{{p}_{i}}^{fixed},{{q}_{j}}^{unfixed}=1-{\alpha }_{{q}_{j}}^{unfixed},\\ 1\le i\le {\rho }_{fixed},1\le j\le {\rho }_{unfixed}\end{array}\},\,({q}_{1},\cdots ,{q}_{{\rho }_{unfixed}})\in I.$$

### Boolean simulations to estimate the attractor landscape of a Boolean network

We performed all Boolean simulations using MATLAB. To find out attractor states of large-scale Boolean networks (CACC49, T-cell54, and MAPK33), we estimated the attractor landscape of each network using a Monte Carlo approach. Each of 10,000,000 randomly selected states was substituted in a deterministic Boolean update rule as an initial state and then the state of each node in a network was synchronously updated following the logical rule. Then we obtained attractor states to which the initial states converge and the approximated ratio of each basin of attraction.

### Boolean simulations to compare temporary versus persistent interventions

One-time temporary perturbation of BRC (Fig. 5b, left) was implemented by flipping the state of a node in a BRC set to the value of the node in the BRC set and tracking the state trajectory from the perturbed state to the desired attractor state. On the other hand, persistent perturbation of conventional control methods (Fig. 5b, right) was implemented by persistently fixing the state of each node in a conventional control set to the value of the node (directly fixed values) in the control set. The nodes and their directly fixed values are placed in the “1^st^ state” in the right of Fig. 5b. Substituting the directly fixed values of all the nodes into the Boolean update rules at time step *t* results in some other fixed node values (indirectly fixed values) at time step *t* + 1, where the nodes of fixed values are placed in the “2^nd^ state”. This process was repeated up to the “8^th^ state”, which is the desired attractor state.

## Supplementary information

Supplementary Information

Supplementary Data 1

Supplementary Data 2

Supplementary Data 3

Supplementary Data 4

## Data Availability

All codes are available from the authors upon request.
