# Supplementary material for: Minimal intervening control of biomolecular networks leading to a desired cellular state
Source: Sci Rep. 2019 Sep 11;9:13124. doi: 10.1038/s41598-019-49571-6 (PMC6739335; doi:10.1038/s41598-019-49571-6)
Supplement: Supplementary file 1 — Supplementary Information [file 41598_2019_49571_MOESM1_ESM.pdf]

Minimal intervening control of biomolecular networks leading to a desired cellular state

Sang-Mok Choo[1§], Sang-Min Park[2§], and Kwang-Hyun Cho[2,*]

[1]Department of Mathematics, University of Ulsan, Ulsan 44610, Republic of Korea

[2]Department of Bio and Brain Engineering, Korea Advanced Institute of Science and Technology (KAIST), Daejeon 34141, Republic of Korea

Supplementary Information

[§] These two authors contributed equally to this study.

[*]Corresponding author. E-mail: ckh@kaist.ac.kr, Phone: +82-42-350-4325, Fax: +82-42-350-4310, Web: http://sbie.kaist.ac.kr/

Supplementary text

Dependence of mHD on the sizes of a network and a basin

To estimate the variance of average mHD as the size of a network increases, update rules are not considered. We assume that a subset B^k of the state space Ω of a given network X of n nodes denotes a desired basin, where the positive integer k denotes the size of B^k. And undesired states are defined as states contained in $\Omega - B^k$. Let $E\left(mHD \middle| B^k\right)$ denote the average mHD of undesired states to B^k. Using the following Theorem 1 and the corresponding simulation, we suggest that the average mHD divided by the network size (called a "normalized average mHD") decreases when the network size increases for basins of the fixed size $\frac{k}{2^n}$ in the state space.

Theorem 1.

Let $B^k = \left\{B_i \in \Omega_1 \middle| 1 \le i \le k\right\}$ for the state space Ω_1 of a network of n nodes and a positive integer k. Let $\widetilde{B}^{2k} = \left\{\widetilde{B}_i \in \Omega_2 \middle| 1 \le i \le 2k\right\}$ for the state space Ω_2 of a network of nodes $n+1$, where $\widetilde{B}_\ell = \left(B_\ell, 0\right)$ and $\widetilde{B}_{k+\ell} = \left(B_\ell, 1\right)$ $\left(1 \le \ell \le k\right)$. Then B^k and $\widetilde{B}^{2k}$ have the same relative size $\frac{k}{2^n}$ and

$$\frac{E\left(mHD \middle| B^k\right)}{n} > \frac{E\left(mHD \middle| \widetilde{B}^{2k}\right)}{n+1}$$

Proof. Let $\widetilde{B}_{zero}=\left\{\widetilde{B}_1,\widetilde{B}_2,\cdots,\widetilde{B}_k\right\}$ and $\widetilde{B}_{one}=\left\{\widetilde{B}_{k+1},\cdots,\widetilde{B}_{2k}\right\}$. Symbols $\tilde{x}_\ell$, $\tilde{x}_{zero,\ell}$ and $\tilde{x}_{one,\ell}$ denote the numbers of states of mHD ℓ to $\widetilde{\widetilde{B}}^{2k}$, $\widetilde{B}_{zero}$ and $\widetilde{B}_{one}$, respectively.

Then for $\tilde{s}\in\Omega_2-\widetilde{\widetilde{B}}^{2k}$

$$\begin{aligned}
&mHD\left(\tilde{s},\widetilde{\widetilde{B}}^{2k}\right)=\ell\\
&\Leftrightarrow mHD\left(\tilde{s},\widetilde{B}_{zero}\right)=\ell \text{ or } mHD\left(\tilde{s},\widetilde{B}_{one}\right)=\ell \qquad ,\\
&\Leftrightarrow \begin{cases} mHD\left(\tilde{s},\widetilde{B}_{zero}\right)=\ell \\ mHD\left(\tilde{s},\widetilde{B}_{one}\right)=\ell+1 \end{cases} \text{or} \begin{cases} mHD\left(\tilde{s},\widetilde{B}_{zero}\right)=\ell+1 \\ mHD\left(\tilde{s},\widetilde{B}_{one}\right)=\ell \end{cases}
\end{aligned}$$

which gives

$$\tilde{x}_\ell=\tilde{x}_{zero,\ell}+\tilde{x}_{one,\ell}\,.\text{---(eq1)}$$

Since there is no state of mHD $n+1$ due to the definition of $\widetilde{B}$, we have

$$\tilde{x}_{n+1}=0\,.\text{ --- (eq2)}$$

Therefore

$$\begin{aligned}
\frac{E\left(mHD\middle|\widetilde{\widetilde{B}}^{2k}\right)}{n+1}&=\frac{1}{n+1}\left\{1\cdot\frac{\tilde{x}_1}{2^{n+1}-2k}+2\cdot\frac{\tilde{x}_2}{2^{n+1}-2k}+\cdots+n\cdot\frac{\tilde{x}_n}{2^{n+1}-2k}+(n+1)\cdot\frac{\tilde{x}_{n+1}}{2^{n+1}-2k}\right\}\\
&=\frac{1}{n+1}\sum_{\ell=1}^{n}\frac{\ell\cdot\tilde{x}_\ell}{2^{n+1}-2k}=\frac{1}{n+1}\sum_{\ell=1}^{n}\frac{\ell\cdot\left(\tilde{x}_{zero,\ell}+\tilde{x}_{\text{one},\ell}\right)}{2^{n+1}-2k}\left\langle\because(eq2),\ (eq1)\right.\\
&=\frac{1}{n+1}\left(\sum_{\ell=1}^{n}\frac{\ell\cdot\tilde{x}_{zero\ell}}{2^{n+1}-2k}+\sum_{\ell=1}^{n}\frac{\ell\cdot\tilde{x}_{\text{one},\ell}}{2^{n+1}-2k}\right)=\frac{1}{n+1}\left(\sum_{\ell=1}^{n}\frac{\ell\cdot x_\ell}{2^{n+1}-2k}+\sum_{\ell=1}^{n}\frac{\ell\cdot x_\ell}{2^{n+1}-2k}\right)\\
&=\frac{1}{n+1}\sum_{\ell=1}^{n}\frac{\ell\cdot x_\ell}{2^n-k}\\
&<\frac{1}{n}\sum_{\ell=1}^{n}\frac{\ell\cdot x_\ell}{2^n-k}=\frac{E\left(mHD\middle|B^k\right)}{n},
\end{aligned}$$

where x_ℓ denotes the number of states of mHD ℓ to B^k.

QED.

Remark 1. In Theorem 1, we proved that the normalized average mHD decreases as the network size increases and the relative size of the desired basins is fixed, where the basins are assumed to have a special structure. We gave a simulation example in Supplementary Fig. 6 to show the property without the restriction on the structure of basins.

Let $E_n^1 = \frac{1}{n} E\left(mHD \middle| B^1\right)$ for a singleton B^1 of a state. Without loss of generality, the singleton B^1 can be assumed to be the set of the zero state $\left(0,\cdots,0\right)$ when calculating $E_n^1 = \frac{1}{n} E\left(mHD \middle| B^1\right)$. Let x_i denote the number of states of mHD i to B^1. Then we have

$$
\begin{aligned}
E_n^1 = \frac{1}{n} E\left(mHD \middle| B^1\right) &= \frac{1}{n} \cdot \frac{x_1}{2^n - 1} + \frac{2}{n} \cdot \frac{x_2}{2^n - 1} + \cdots + \frac{n}{n} \cdot \frac{x_n}{2^n - 1} \\
&= \frac{1}{n} \cdot \frac{\sum_{i=1}^{n} i \cdot {}_n C_i}{2^n - 1} \\
&= \frac{1}{n} \cdot \frac{n \cdot 2^{n-1}}{2^n - 1} = \frac{2^{n-1}}{2^n - 1}
\end{aligned}
$$

Therefore the normalized average mHD to a singleton converges to 1/2 as n goes to infinity.

Theorem 2. Let B be a singleton of a state of zero state values in a network of n nodes. Then

$$
E_n^1 = \frac{1}{n} E\left(mHD \middle| B^1\right) = \frac{2^{n-1}}{2^n - 1}
$$

Using the following theorems and the corresponding simulations, we suggest that the normalized average mHD decreases when the network size is fixed and two states in B are apart from each other.

Theorem3. Let B^2 be the set $\{B_0, B_1\}$ of two states in a network of n nodes, where the state values of nodes in $B_0 = (0,\cdots,0)$ are zero and $B_1 = (0,\cdots,0,1)$ has value 0 of all nodes except for the last node. Let $E_n^{2,HD1} = \frac{1}{n} E(mHD|B^2)$. Then

$$E_n^{2,HD1} = \frac{n-1}{n} \cdot \frac{2^{n-2}}{2^{n-1}-1}$$

Proof. Let Ω be the state space of the given network in the theorem. Let $d_i = \left|\{s \in B^2 | HD(s, B_0) = i\}\right|$ $(1 \le i \le n)$, where symbol $|A|$ denotes the number of elements of a set A and $HD(s, \tilde{s})$ is the Hamming distance between states s and $\tilde{s}$. Using the definition of $B^2 = \{B_0, B_1\}$, we have $d_1 = 1$ and $d_\ell = 0$ $(2 \le \ell \le n)$. Let $S_k^i = \left|\{s \in \Omega | \mathrm{HD}(s, B_0) = i \text{ and } \mathrm{HD}(s, B_1) = k\}\right|$ $(2 \le i \le n,\ 1 \le k \le i-1)$, where $\mathrm{HD}(s, B_0) = i$ implies that the number of values 1 in the state s is i and $\mathrm{HD}(s, B_1) = k$ implies that the number of values 1 in s is either $k-1$ or $k+1$. Then

$$i = k+1,\ S_k^i = \begin{cases} S_{i-1}^i & (k = i-1) \\ 0 & (1 \le k \le i-2) \end{cases} \text{ and } S_{i-1}^i = {}_{n-1}C_{i-1},$$

which gives the desired result

$$
\begin{aligned}
E_n^{2,HD1} &= \frac{1}{n}\cdot\frac{1}{2^n-2}\left[\sum_{i=1}^{n} i\cdot {}_nC_i - \sum_{i=1}^{n} i\cdot d_i - \sum_{i=2}^{n}\sum_{k=1}^{i-1}(i-k)\cdot S_k^i\right] \\
&= \frac{1}{n}\cdot\frac{1}{2^n-2}\left[n\cdot 2^{n-1} - \sum_{i=1}^{n} i\cdot d_i - \sum_{i=2}^{n}\left\{(i-(i-1))\cdot S_{i-1}^i + \sum_{k=1}^{i-2}(i-k)\cdot S_k^i\right\}\right] \\
&= \frac{1}{n}\cdot\frac{1}{2^n-2}\left[n\cdot 2^{n-1} - 1\cdot d_1 - \sum_{i=2}^{n} 1\cdot S_{i-1}^i\right] \\
&= \frac{1}{n}\cdot\frac{1}{2^n-2}\left(n\cdot 2^{n-1} - 1 - \sum_{i=2}^{n} {}_{n-1}C_{i-1}\right) = \frac{1}{n}\cdot\frac{1}{2^n-2}\left(n\cdot 2^{n-1} - 2^{n-1}\right) \\
&= \frac{n-1}{n}\cdot\frac{2^{n-1}}{2^n-2} = \frac{n-1}{n}\cdot\frac{2^{n-2}}{2^{n-1}-1}
\end{aligned}
.
$$

QED.

Remark 2. Using Theorems 2 and 3, we obtained

$$E_n^1 = \frac{2^{n-1}}{2^n-1} > E_{n+1}^{2,HD1} = \frac{n}{n+1}\cdot\frac{2^{n-1}}{2^n-1}$$

and

$$nE_n^1 = E\left(mHD \middle| B^1\right) = \frac{n2^{n-1}}{2^n-1} = (n+1)E_{n+1}^{2,HD1}.$$

In particular, the last equality in the proof of Theorem 1 implies that the average mHDs for desired basins of a fixed relative size does not increase even if the network size increases.

Theorem4. Let B^2 be the set $\{B_0, B_1\}$ of two states in a network of n nodes, where the state values of nodes in $B_0 = (0,\cdots,0)$ are zero and $B_1 = (0,\cdots,0,1,1)$ has value 0 of all nodes except for the last two nodes. Let $E_n^{2,HD2} = \frac{1}{n}E\left(mHD \middle| B^2\right)$. Then

$$E_n^{2,HD2} = \frac{n-1}{n}\cdot\frac{2^{n-2}}{2^{n-1}-1}$$

Proof. Let Ω be the state space of the given network in the theorem. Let $d_i = \left|\left\{ s \in B^2 \middle| HD(s,B_0) = i \right\}\right|$ $(1 \le i \le n)$, where symbol $|A|$ denotes the number of elements of a set A and $HD(s,\tilde{s})$ is the Hamming distance between states s and $\tilde{s}$. Using the definition of $B^2 = \{B_0, B_1\}$, we have $d_2 = 1$ and $d_\ell = 0 (1 \le \ell \le n,\ \ell \ne 2)$. Let $S_k^i = \left|\left\{ s \in \Omega \middle| \mathrm{HD}(s,B_0) = i \text{ and } \mathrm{HD}(s,B_1) = k \right\}\right|$ $(2 \le i \le n,\ 1 \le k \le i-1)$, where $\mathrm{HD}(s,B_0) = i$ implies that the number of values 1 in s is i and $\mathrm{HD}(s,B_1) = k$ implies that the number of values 1 in s is $k-2$, k or $k+2$. Then

$$i = k + 2,\ S_k^i = \begin{cases} S_{i-2}^i & (k = i-2) \\ 0 & (others) \end{cases} \text{ and } S_{i-2}^i = {}_{n-2}C_{i-2},$$

which gives the desired result

$$\begin{aligned}
E_n^{2,HD2} &= \frac{1}{n} \cdot \frac{1}{2^n - 2} \left[\sum_{i=1}^{n} i \cdot {}_nC_i - \sum_{i=1}^{n} i \cdot d_i - \sum_{i=2}^{n} \sum_{k=1}^{i-1} (i-k) \cdot S_k^i \right] \\
&= \frac{1}{n} \cdot \frac{1}{2^n - 2} \left[n \cdot 2^{n-1} - \sum_{i=1}^{n} i \cdot d_i - \sum_{i=2}^{n} \left\{ (i - (i-2)) \cdot S_{i-2}^i + \sum_{1 \le k \le i-1, k \ne i-2} (i-k) \cdot S_k^i \right\} \right] \\
&= \frac{1}{n} \cdot \frac{1}{2^n - 2} \left[n \cdot 2^{n-1} - 2 \cdot d_2 - \sum_{i=2}^{n} 2 \cdot S_k^{k+2} \right] \\
&= \frac{1}{n} \cdot \frac{1}{2^n - 2} \left(n \cdot 2^{n-1} - 2 - \sum_{i=2}^{n} 2 \cdot {}_{n-2}C_{i-2} \right) = \frac{1}{n} \cdot \frac{1}{2^n - 2} \left(n \cdot 2^{n-1} - 2 - 2 \cdot \left(2^{n-2} - 1 \right) \right) \\
&= \frac{1}{n} \cdot \frac{n \cdot 2^{n-1} - 2^{n-1}}{2^n - 2} = \frac{n-1}{n} \cdot \frac{2^{n-2}}{2^{n-1} - 1}
\end{aligned}.$$

QED.

Remark 3. Using Theorems 3 and 4, we have

$$E_n^{2,HD1} = E_n^{2,HD2},$$

and using Remark 2 together, we have

$$E\left(mHD \middle| B^1\right) = \left(n+1\right) E_{n+1}^{2,HD1} = \left(n+1\right) E_{n+1}^{2,HD2},$$

which implies that the average mHDs for desired basins of a fixed relative size does not increase even if the network size increases.

Theorem5. Let B^2 be the set $\{B_0, B_1\}$ of two states in a network of n nodes, where the state values of nodes in $B_0 = (0,\cdots,0)$ are zero and $B_1 = (0,\cdots,0,1,1,1)$ has value 0 of all nodes except for the last three nodes. Let $E_n^{2,HD3} = \frac{1}{n} E\left(mHD \middle| B^2\right)$. Then

$$E_n^{2,HD3} = \frac{1}{n} \cdot \frac{(n-1)\cdot 2^{n-1} - \left(2^{n-2}+3\right)}{2^n - 2}$$

Proof. Let Ω be the state space of the given network in the theorem. Let $d_i = \left|\left\{ s \in B^2 \middle| HD(s,B_0) = i \right\}\right| \; (1 \le i \le n)$, , where symbol $|A|$ denotes the number of elements of a set A and $HD\left(s,\tilde{s}\right)$ is the Hamming distance between states s and $\tilde{s}$. Using the definition of $B^2 = \{B_0, B_1\}$, we have $d_3 = 1$ and $d_\ell = 0\left(1 \le \ell \le n,\; \ell \ne 3\right)$. Let $S_k^i = \left|\left\{ s \in \Omega \middle| \mathrm{HD}(s,B_0) = i \text{ and } \mathrm{HD}(s,B_1) = k \right\}\right| \quad \left(2 \le i \le n,\; 1 \le k \le i-1\right)$, where $\mathrm{HD}(s,B_0) = i$ implies that the number of values 1 in s is i and $\mathrm{HD}(s,B_1) = k$ implies that the number of values 1 in s is $k-3$, $k-1$, $k+1$ or $k+3$. Then

$$i = k+1,\ k+3,\ S_k^i = \begin{cases} S_{i-1}^i & (k = i-1) \\ S_{i-3}^i & (k = i-3) \\ 0 & (others) \end{cases} \text{ and } \begin{matrix} S_{i-1}^i = {}_3C_1 \cdot {}_{n-3}C_{i-2} = 3 \cdot {}_{n-3}C_{i-2} \\ S_{i-3}^i = {}_3C_0 \cdot {}_{n-3}C_{i-3} = {}_{n-3}C_{i-3} \end{matrix},$$

which gives the desired result

$$\begin{aligned}
E_n^{2,HD3} &= \frac{1}{n} \cdot \frac{1}{2^n - 2} \left[\sum_{i=1}^{n} i \cdot {}_nC_i - \sum_{i=1}^{n} i \cdot d_i - \sum_{i=2}^{n} \sum_{k=1}^{i-1} (i-k) \cdot S_k^i \right] \\
&= \frac{1}{n} \cdot \frac{1}{2^n - 2} \left[\begin{matrix} n \cdot 2^{n-1} - \sum_{i=1}^{n} i \cdot d_i \\ -\sum_{i=2}^{n} \left\{ \left(i - (i-1)\right) \cdot S_{i-1}^i + \left(i - (i-3)\right) \cdot S_{i-3}^i \right\} \end{matrix} \right] \\
&= \frac{1}{n} \cdot \frac{1}{2^n - 2} \left[n \cdot 2^{n-1} - 3 \cdot d_3 - \left(\sum_{i=2}^{n} S_{i-1}^i + \sum_{i=3}^{n} 3 \cdot S_{i-3}^i \right) \right] \\
&= \frac{1}{n} \cdot \frac{1}{2^n - 2} \left[n \cdot 2^{n-1} - 3 - \left(\sum_{i=2}^{n} 3 \cdot {}_{n-3}C_{i-2} + \sum_{i=3}^{n} 3 \cdot {}_{n-3}C_{i-3} \right) \right] \\
&= \frac{1}{n} \cdot \frac{1}{2^n - 2} \left[n \cdot 2^{n-1} - 3 - \left(3\left(2^{n-3} - 1\right) + 3\left(2^{n-3} - 1\right) \right) \right] \\
&= \frac{1}{n} \cdot \frac{n \cdot 2^{n-1} - 3 \cdot 2^{n-2} + 3}{2^n - 2} = \frac{1}{n} \cdot \frac{(n-1) \cdot 2^{n-1} - \left(2^{n-2} + 3\right)}{2^n - 2}
\end{aligned}$$

QED.

Remark 4. Using Theorems 1, 2, 3, 4 and 5, we suggest that the normalized average mHD has the following upper bound

$$\frac{E\left(mHD \middle| B^k\right)}{n} \le \frac{1}{\ell} E\left(mHD \middle| B^1\right) = E_\ell^1 = \frac{2^{\ell-1}}{2^{\ell-2} - 1} \quad \forall n \ge \ell \ge 3, \forall k \ge 1,$$

where the property is presented by a simulation example in Supplementary Fig. 7.

Remark 5. The inequality

$$nE_n^{2,HD2} = (n-1)\cdot\frac{2^{n-2}}{2^{n-1}-1} > nE_n^{2,HD3} = \frac{(n-1)2^{n-1}-(2^{n-2}+3)}{2^n-2}$$

is equivalent to

$$\begin{aligned}(n-1)\cdot\frac{2^{n-2}}{2^{n-1}-1} &> \frac{(n-1)2^{n-1}-(2^{n-2}+3)}{2^n-2}\\ &= (n-1)\cdot\frac{2^{n-1}-\frac{1}{n-1}(2^{n-2}+3)}{2^n-2}\\ &= (n-1)\cdot\frac{2^{n-2}-\frac{1}{2(n-1)}(2^{n-2}+3)}{2^{n-1}-1}\end{aligned},$$

and then we obtain

$$nE_n^{2,HD2} > nE_n^{2,HD3}.$$

So, we suggest that the average mHD decreases for any fixed network size when the two states in B^k are farther apart from each other (see Supplementary Fig. 8). Therefore, using Remarks 2 and 3 together, we obtain

$$nE_n^1 = (n+1)E_{n+1}^{2,HD1} = (n+1)E_{n+1}^{2,HD2} > (n+1)E_{n+1}^{2,HD3},$$

and then we suggest that the average mHDs for desired basins of a fixed relative size does not increase even if the network size increases (see Supplementary Fig. 8).

Extension of BRC to a homogeneous system of finite linear differential equations with constant coefficients

The general solution of any homogeneous system of finite linear ordinary differential equations (ODEs) with constant coefficients can in general be explicitly written in a closed form. Using the closed-form solution, we can identify an exact basin and so apply the concept of BRC to the system. This process is explained in the following steps with an example.

Step 1. Construction of the general solution in a closed form

Consider a network of three nodes whose dynamics are modelled by the homogeneous system of three linear ordinary differential equations with constant coefficients

$$\frac{d}{dt}X(t) = AX(t) \ (t \geq 0),$$

where $X(t) = [x_1(t) \ x_2(t) \ x_3(t)]^T$ is the transpose of the vector of $x_i(t)$ and

$$A = \begin{bmatrix} 1 & -3 & 2 \\ 1 & 0 & 1 \\ 3 & -2 & 2 \end{bmatrix}.$$

Symbol $x_i(t)$ $(i = 1,2,3)$ denotes the state of the ith node at time t. Matrix A has three eigenvalues $-1,\ 2+i,\ 2-i$ and their corresponding eigenvectors

$$V_1 = [-1 \ 0 \ 1]^T, V_2 = [-1 \quad -1+i \quad -2+i]^T, V_3 = [-1 \quad -1-i \quad -2-i]^T,$$

where i denotes the imaginary number. Then the general solution $X(t)$ is written as follows: for generic constants $C_i (1 \le i \le 3)$

$$X(t) = C_1 e^{-t} V_1 + C_2 e^{2t} (\cos t V_a - \sin t V_b) + C_3 e^{2t} (\sin t V_a + \cos t V_b), \text{ --- (1)}$$

where V_a and V_b are real and imaginary parts of V_2 such that

$$V_a = [-1 \ \ -1 \ \ -2]^T \text{ and } V_b = [0 \ \ 1 \ \ 1]^T.$$

For the application of BRC we write the general solution $X(t)$ in (1) as follows:

$$\begin{aligned} x_1(t) &= -C_1 e^{-t} - C_2 e^{2t} \cos t - C_3 e^{2t} \sin t \\ x_2(t) &= C_2 e^{2t} (-\cos t - \sin t) + C_3 e^{2t} (-\sin t + \cos t) \qquad \text{--- (2)} \\ x_3(t) &= C_1 e^{-t} + C_2 e^{2t} (-2\cos t - \sin t) + C_3 e^{2t} (-2\sin t + \cos t) \end{aligned}$$

Step 2. Identification of the exact basin of a desired attractor

We assume that a desired point attractor state is $(x_1, x_2, x_3) = (0,0,0)$. Using the general solution (2), we have the exact basin

$$\{(r, 0, -r) | r \in R\} \text{ --- (3)}$$

since the exact basin of the desired attractor is defined as the set of all initial conditions under which the ODE system is convergent to the desired attractor, where R denotes the set of real numbers.

Step 3. Determination of an undesired state

The undesired states are any initial state under which the ODE system does not converge to the desired attractor. Letting an initial condition be

$$X(0) = C_1V_1 + C_2V_a + C_3V_b = [-0.5 \quad 0 \quad -0.5]^T \quad (C_1 = 0, C_2 = C_3 = 0.5),$$

the ODE system with the initial condition $X(0) = [-0.5 \quad 0 \quad -0.5]^T$ is divergent. So, an undesired state can be the state

$$(x_1, x_2, x_3) = (-0.5, 0, -0.5).$$

Step 4. Identifications of control target sets and boundary states

We assume that a given control strategy is to identify the minimum number of control target nodes to be perturbed which drive the undesired state into the exact basin (3). If x_1 is perturbed to 0.5 or x_2 is perturbed to 0.5, then the perturbed state can be contained in the exact basin. Therefore minimum control target sets are

$$\{x_1 = 0.5\} \text{ and } \{x_2 = 0.5\},$$

which result in the two boundary states from the undesired state

$$(x_1, x_2, x_3) = (-0.5, 0, 0.5) \text{ and } (x_1, x_2, x_3) = (0.5, 0, -0.5)$$

and the boundary of the exact basin from the undesired state

$$\{(x_1, x_2, x_3) = (-0.5, 0, -0.5),\ (x_1, x_2, x_3) = (0.5, 0, -0.5)\}.$$

Supplementary Figure 1

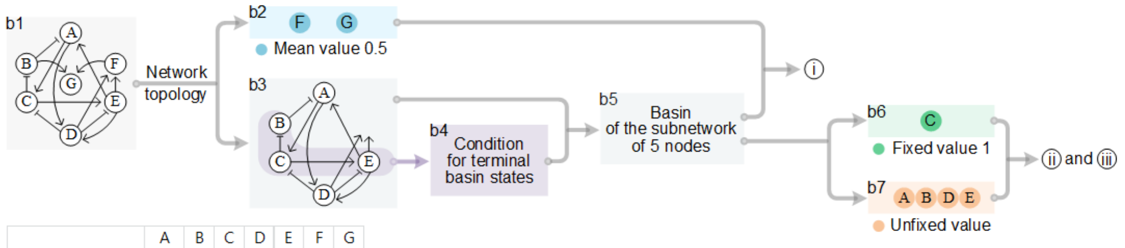

	A	B	C	D	E	F	G
Desired attractor β	1	0	1	1	1	1	0
Undesired state α	0	0	0	0	1	0	0

b1

A* = ¬B ∧ E
B* = ¬C
C*= A ∨ ¬D
D*= A ∨ E
E*= C ∨ ¬D
F* = D ∧ E
G* =B ∧ F

b3

A* = ¬B ∧ E
B* = ¬C
C*= A ∨ ¬D
D*= A ∨ E
E*= C ∨ ¬D

b4

E*= ¬B* ∨ ¬D

(B*, E*)=(0,1)

b5

	A	B	C	D	E
state 1	0	0	1	0	0
state 2	0	0	1	0	1
state 3	0	1	1	0	0
state 4	1	0	1	0	1
state 5	1	0	1	1	1

b6 and b7

	A	B	C	D	E
state 1	0	0	1	0	0
state 2	0	0	1	0	1
state 3	0	1	1	0	0
state 4	1	0	1	0	1
state 5	1	0	1	1	1

ⓘ Identify the exact basin of attraction to β

	State																			
	1	2	3	4	5	6	7	8	9	10	11	12	13	14	15	16	17	18	19	20
A	0	0	0	0	0	0	1	1	1	1	0	0	0	0	0	0	1	1	1	1
B	0	0	0	0	1	1	0	0	0	0	0	0	0	0	1	1	0	0	0	0
C	1	1	1	1	1	1	1	1	1	1	1	1	1	1	1	1	1	1	1	1
D	0	0	0	0	0	0	0	0	1	1	0	0	0	0	0	0	0	0	1	1
E	0	0	1	1	0	0	1	1	1	1	0	0	1	1	0	0	1	1	1	1
F	0	1	0	1	0	1	0	1	0	1	0	1	0	1	0	1	0	1	0	1
G	0	0	0	0	0	0	0	0	0	0	1	1	1	1	1	1	1	1	1	1

ⓘⓘ Identify the boundary of the basin from α : { (A,B,C,D,E,F,G) = (0,0,1,0,1,0,0) }

ⓘⓘⓘ Determine a minimum control target set : { C = 1}

Supplementary Figure 1. Update rules and data used in Fig. 1b. The first row (upper b1 to upper b7) is explained in the caption of Fig. 1b in the main text. Lower b1 denotes the Boolean update rules for the original network in upper b1, where symbol "*" of a node on the left side of each equation denotes its value at time step t+1 and a node on the right side of the equation denotes its value at time step t. The symbols "¬", "∨" and "∧" denote the logic operators "NOT", "OR" and "AND". The reduced network of nodes A, B, C, D and E in upper b3 is obtained from the original network by removing

symmetric nodes F and G, which has the reduced attractor state (A, B, C, D, E) = (1, 0, 1, 1, 1) obtained from desired attractor β and the reduced update rules in lower b3. The basin of the reduced attractor is given in lower b5, which are hierarchically identified by following the process presented in the main text by using (1) the reduced attractor, (2) the reduced update rules and (3) sufficient conditions for terminal basin states in lower b4. To clearly and generally explain this process, an example network of 5 nodes with a desired attractor of length 2 is used in Supplementary Fig. 3. The table in ⓘ is obtained by concatinating each state in lower b5 and four possible states (F, G) = (0, 0), (1, 0), (0, 1) , (1, 1). For example, there exist four states (A, B, C, D, E, F, G) = (0, 0, 1, 0, 0, 0, 0), (0, 0, 1, 0, 0, 1, 0), (0, 0, 1, 0, 0, 0, 1), (0, 0, 1, 0, 0, 1, 1), which are states 1, 2, 11 and 12 in the table in ⓘ. The ordering of states in the table shows that node G is symmetric. Symmetric structure of F and G are presented in Supplementary Fig. 2. HDs from α to each basin state are 2, 3, 1, 2, 3, 4, 2, 3, 3, 4, 3, 4, 2, 3, 4, 5, 3, 4, 4 and 5, leading to mHD = 1 only for basin state 3. So, state 3 becomes a unique boundary state from α and the boundary of the basin from α is the singleton of state 3 {(A, B, C, D, E, F, G) = (0, 0, 1, 0, 1, 0, 0)}, leading to an unique minimum control target set is {C=0}.

Supplementary Figure 2

State	A	B	C	D	E	F	G
1	0	0	1	0	0	0	0
2	0	0	1	0	0	1	0
3	0	0	1	0	1	0	0
4	0	0	1	0	1	1	0
5	0	1	1	0	0	0	0
6	0	1	1	0	0	1	0
7	1	0	1	0	1	0	0
8	1	0	1	0	1	1	0
9	1	0	1	1	1	0	0
10	1	0	1	1	1	1	0
11	0	0	1	0	0	0	1
12	0	0	1	0	0	1	1
13	0	0	1	0	1	0	1
14	0	0	1	0	1	1	1
15	0	1	1	0	0	0	1
16	0	1	1	0	0	1	1
17	1	0	1	0	1	0	1
18	1	0	1	0	1	1	1
19	1	0	1	1	1	0	1
20	1	0	1	1	1	1	1

State	A	B	C	D	E	F	G
1	0	0	1	0	0	0	0
3	0	0	1	0	1	0	0
5	0	1	1	0	0	0	0
7	1	0	1	0	1	0	0
9	1	0	1	1	1	0	0
11	0	0	1	0	0	0	1
13	0	0	1	0	1	0	1
15	0	1	1	0	0	0	1
17	1	0	1	0	1	0	1
19	1	0	1	1	1	0	1
2	0	0	1	0	0	1	0
4	0	0	1	0	1	1	0
6	0	1	1	0	0	1	0
8	1	0	1	0	1	1	0
10	1	0	1	1	1	1	0
12	0	0	1	0	0	1	1
14	0	0	1	0	1	1	1
16	0	1	1	0	0	1	1
18	1	0	1	0	1	1	1
20	1	0	1	1	1	1	1

State	A	B	C	D	E	F	G
1	0	0	1	0	0	0	0
11	0	0	1	0	0	0	1
2	0	0	1	0	0	1	0
12	0	0	1	0	0	1	1
3	0	0	1	0	1	0	0
13	0	0	1	0	1	0	1
4	0	0	1	0	1	1	0
14	0	0	1	0	1	1	1
5	0	1	1	0	0	0	0
15	0	1	1	0	0	0	1
6	0	1	1	0	0	1	0
16	0	1	1	0	0	1	1
7	1	0	1	0	1	0	0
17	1	0	1	0	1	0	1
8	1	0	1	0	1	1	0
18	1	0	1	0	1	1	1
9	1	0	1	1	1	0	0
19	1	0	1	1	1	0	1
10	1	0	1	1	1	1	0
20	1	0	1	1	1	1	1

Supplementary Figure 2. Symmetric nodes F and G of mean value 0.5 in Fig. 1b.

The three sets of 20 states on the right side, the center and the left side are equal and each set is the basin of attractor β in Supplementary Fig. 1. The two sets on the left side and the center show that nodes G and F are symmetric of mean value 0.5 in the basin, respectively. The right side shows the symmetric structure of (F, G).

Supplementary Figure 3

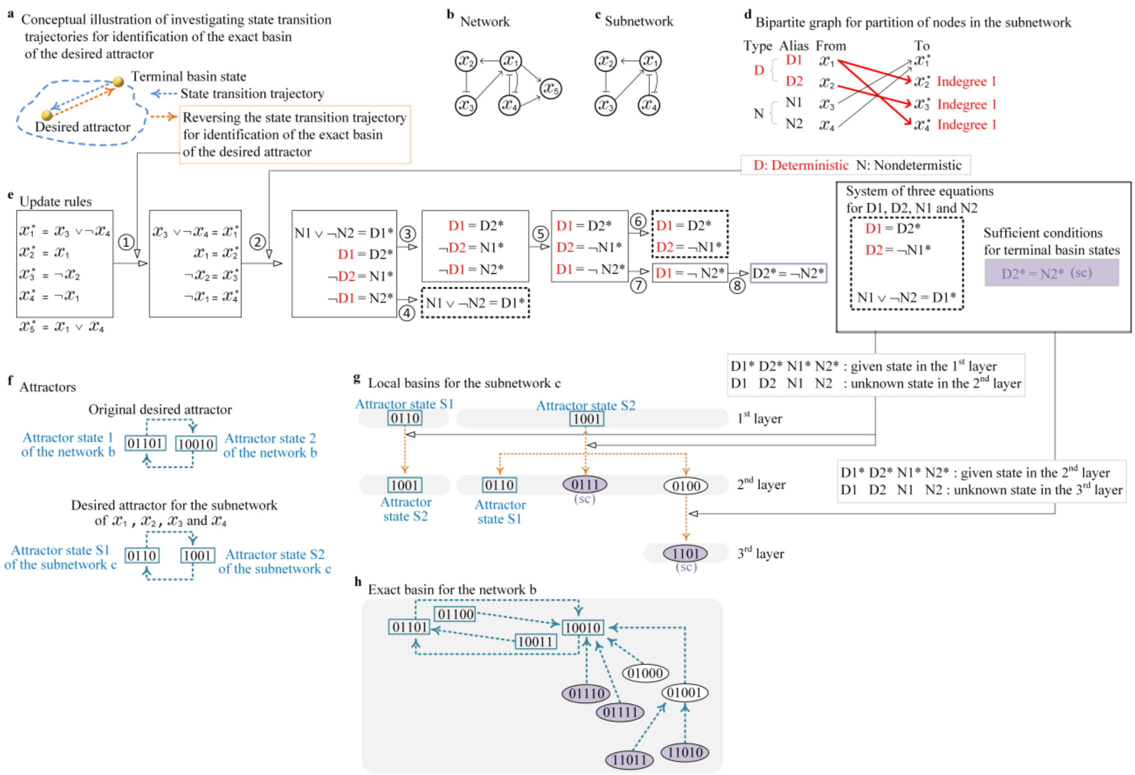

Supplementary Figure 3. Identification process of the exact basin with an example network. a Conceptual illustratation of identifying basin states with respect to the direction of state transition trajectory. The dotted closed curve denotes the set of terminal basin states converging to the desired attractor. **b** The toy network of five nodes. Arrows and bar-headed lines in the network represent activation and inhibition, respectively. **c** The subnetwork obtained from the network **b** by removing the symmetric node x_5. **d** Classification of nodes. “Indegree 1” of x_2^* means that there is only one input node to x_2^*. “From x_1 $\rightarrow$ To x_2^*” means that the state value of x_1 at time step t is an input value to the state value of x_2^* at time step $t+1$. Node x_1 is

called “Deterministic” since x_1 is only one input node to x_2^* and each basin state is identified following the reverse of the state trajectory. A deterministic node is denoted by “D”. A “nondeterministic” node means that the node is not deterministic and is denoted by “N”. **e** Transformation nodes and the update rules into (1) symmetric nodes, (2) equations for the other nodes and (3) sufficient conditions for terminal basin states. The Boolean eqautions are the update rules for the network **b**. The update rule for symmetric node x_5 is located outside the box and the update rules for the subnetwork **b** are located inside the box. ① denotes the reversion of the update rules. ② denotes the replacements of node symbol x with “D” and “N” in **d**. ③ denotes the system of equations for determinitic nodes D1 and D2, where D2*, N1* and N2* are given state values. ④ denotes the equation for nondeterminitic nodes N1 and N2, where D1* is a given state value. The dotted box means that no more modification to the equation in the dotted box is given. ⑤ denotes the removal of logic operators in front of D1 and D2. Since there exist two equations for D1, we divided the system of three equations for D1 and D2 into two subsystems. One consists of two equations for D1 and D2, which is denoted by ⑥ and the second consists of the other equation for D1 denoted by ⑦. Using the two equations for D1 in ⑥ and ⑦, we obtained the equation denoted by ⑧ which yields the sufficient conditions (sc) for terminal basin states in the bold box. The

sufficient conditions mean that (D1*, D2*, N1*, N2*) = (·, 1, ·, 1) and (·, 0, ·, 0) are terminal basin states, where the centered dot (·) denotes two values 0 or 1. Using the system of equations for D1, D2, N1, D2 and the sufficient conditions, we identitfied the states (D1, D2, N1, N2) given a state (D1*, D2*, N1*, N2*). **f** Origian and reduced attractors. The top represents the orignal desired attractor of length 2 for the orignal network **b** and the bottom represents the reduced attractor of the original attractor in the subnetwork **c**. **g** Basin of the reduced desred attractor. The desired basin for the subnetwork consists of two local basins which are identified independently. Each attractor is located at the 1st layer of its local basin. Given a state in the 1st layer, states in the 2nd layer are calculated by using the equations in the bold box at the end of **e**. **h** Exact basin of the origianl attractor. Concatination of states in **g** and state values 0 and 1 of symmetric node x_5 yields the exact basin for the network **b**. When using the exact basin in BRC, the state trajectories (dotted arrows) are not used.

Supplementary Figure 4

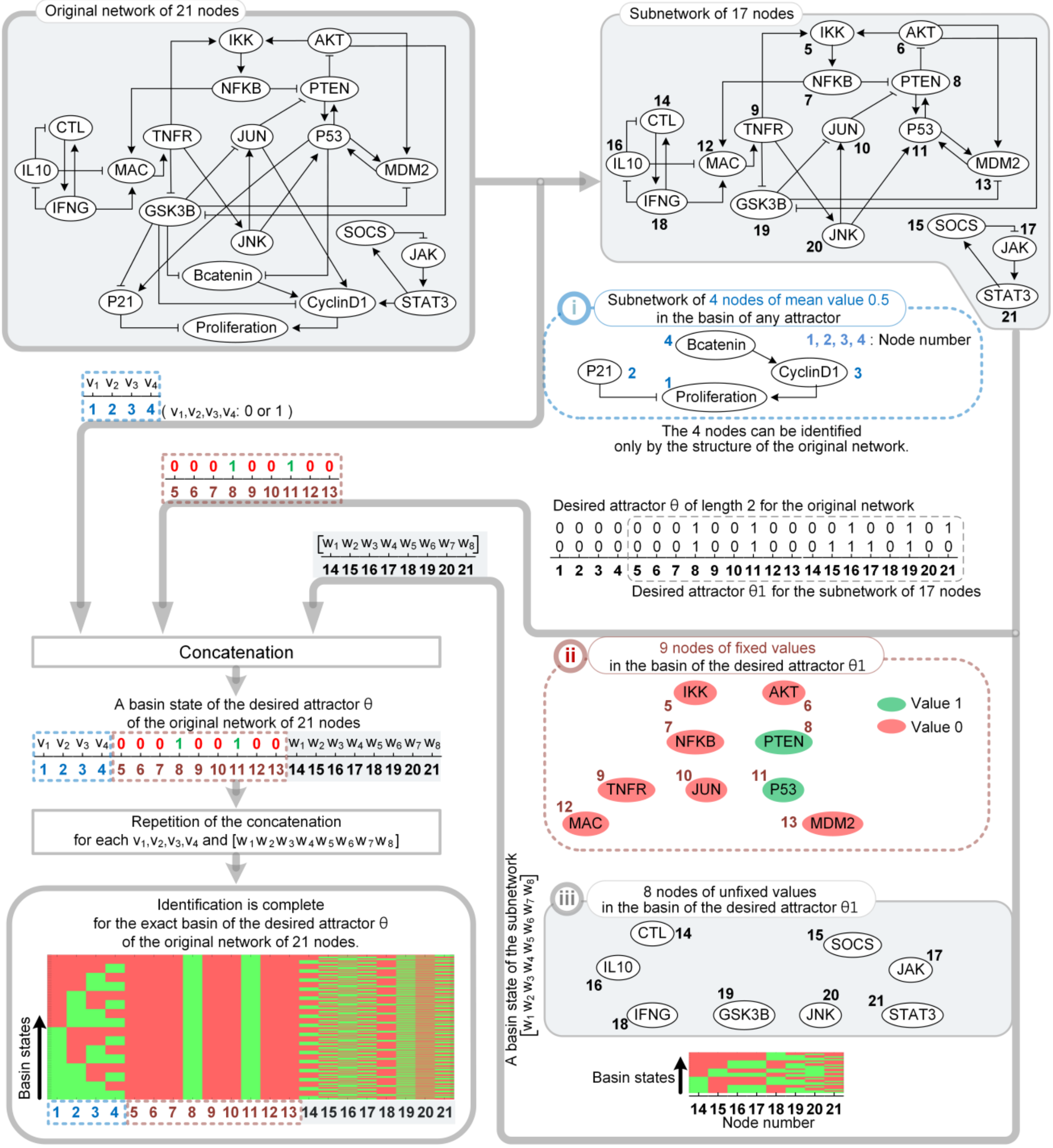

Supplementary Figure 4. Identification of the basin of the attractor θ of CACC21 in Fig. 2a. The basin identification follows the process in Supplementary Fig. 3. The subnetwork of 4 nodes in the blue dotted box (ⓘ) denotes the hierarchical structure of symmetric nodes: Proliferation is the unique symmetric node in CACC21; P21 and

CyclinD1 are symmetric in CACC21 without Proliferation; Bcatenin is symmetric in CACC21 without Proliferation, P21 and CyclinD1. There is no symmetric node in CACC21 without Proliferation, P21, CyclinD1 and Bcatenin. The update rules for CACC21 are in Supplementary Data 1. The desired cyclic attractor state θ of CACC21 is located just below the hierarchical subnetwork of 4 symmetric nodes, where the reduced attractor state θ1 of the subnetwork of 17 nodes is marked with the black dotted box. 9 nodes of fixed values in the basin of θ1 are in the red dotted box (ⓘ) and the remaining 8 nodes are in the grey box (ⓘ), where the fixed values are (IKK, AKT, NFKB, PTEN, TNFR, JUN, P53, MAC, MDM2) = (0,0,0,1,0,0,1,0,0) and state values of nodes 14 to 21 are denoted by nodes w1 to w8. The heatmap in bottom right denotes basin states [w_1, w_2, w_3, w_4, w_5, w_6, w_7, w_8]. On the left side the three parts (values of symmetric nodes in ⓘ, fixed values of 9 nodes in ⓘ and states of 8 nodes in ⓘ) are concatenated, which results in the exact basin in bottom left (sorted to the symmetric nodes, followed by nodes of fixed values).

Supplementary Figure 5

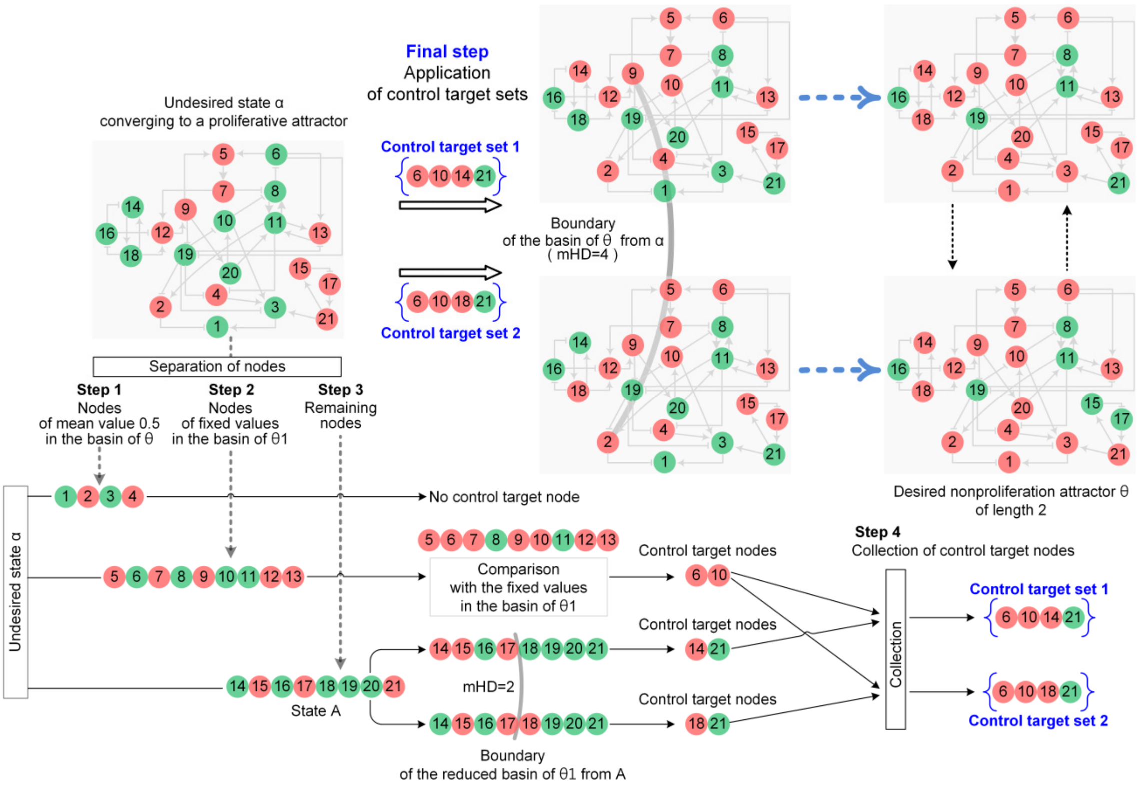

Supplementary Figure 5. Identification process of control target sets in Fig. 2b. The update rules for CACC21 are in Supplementary Data 1 and the attractors θ and θ1 are the same attractors in Supplementary Fig. 4. Green and red colors denote state values 1 and 0, respectively. The process from "Step 1" to "Step 3" is to separate all nodes of CACC21. Step 1 is to find symmetric nodes, which are nodes 1, 2, 3 and 4, where only the network structure is used without using the update rules. "Step 2" is to find nodes of fixed values in the basin of θ1 for the subnetwork of the 17 nodes, which are nodes 5 to 13. Their fixed values are located just above the boxed words "Comparison with the fixed values in the basin of θ1". Using the comparison of the values of nodes 5 to 13 in

α with the fixed values, the values of nodes 6 and 10 must be changed to value 0 ("control target nodes"), which is represented on the right side of the boxed words. "Step 3" is to find nodes of unfixed values in the basin of θ1 which values in α are denoted by "State A". The mHD of state A to the boundary of the reduced basin of θ1 is equal to 2, where the reduced basin of θ1 is obtained by removing the nodes 5 to 13 from the basin of θ1 and state A can be driven to the boundary by using {(node 14, node 21) = (0,1)} ("control target nodes") or {(node 18, node 21) = (0,1)} ("control target nodes"). "Step 4" is to collect control target nodes, which results in two control target sets of α, {(node 6, node 10, node 14, node 21) = (0,0,0,1)} and {(node 6, node 10, node 18, node 21) = (0,0,0,1)}. Therefore perturbation of at least four nodes (mHD = 4) is needed to drive the undesired state α to the basin of θ in BRC. "Final step" is to use a control target set to drive α to the boundary of the basin of θ, after which the state transition flow diagram is presented in top right.

Supplementary Figure 6

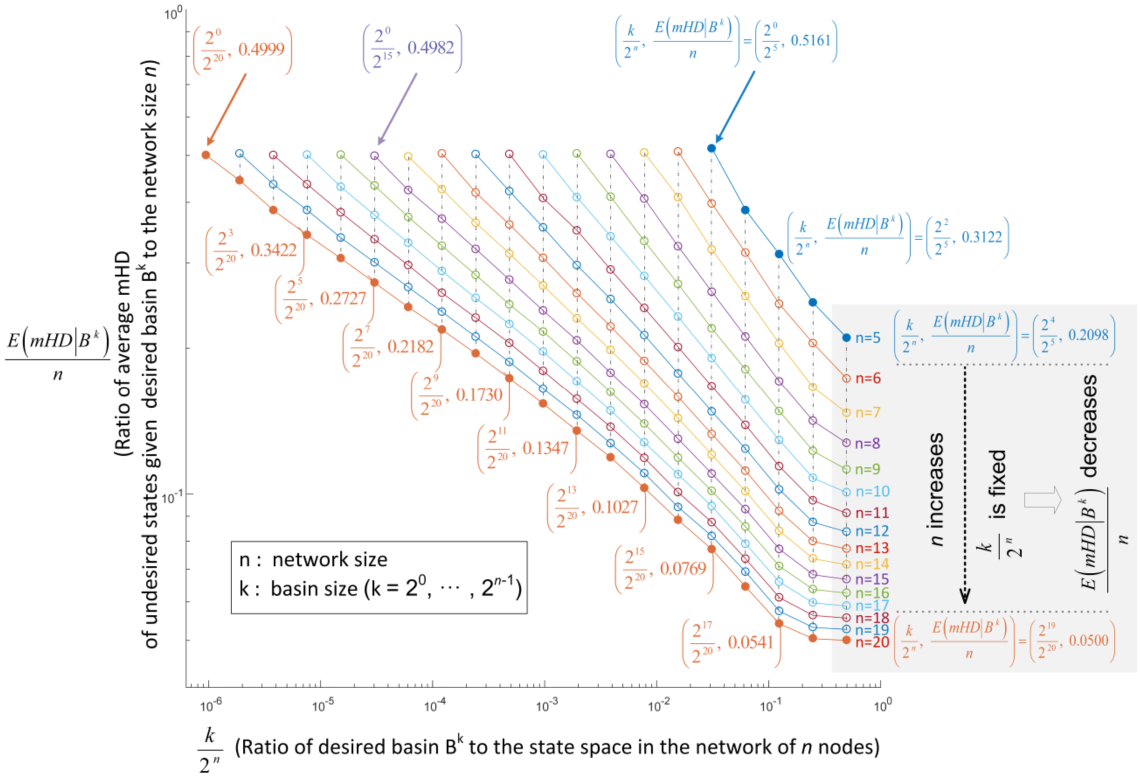

Supplementary Figure 6. Decrease of normalized average mHD as the network size increases and the relative size of desired basins is fixed. A subset B^k of the state space Ω of a given network X of n nodes denotes a desired basin, where the positive integer k denotes the size of B^k. An undesired state is defined as a state contained in $\Omega - B^k$. Symbol $E\left(mHD\middle|B^k\right)$ denotes the average mHD of undesired states to B^k. The 16 points (circles) in the grey box are connected by a dotted vertical line, on which the first and last points are obtained from networks of sizes $n = 5$ and $n = 20$, respectively, and have the coordinates $\left(\frac{k}{2^n}, \frac{E\left(mHD\middle|B^k\right)}{n}\right) = \left(\frac{2^4}{2^5}, 0.2098\right)$

and $\left(\frac{k}{2^n}, \frac{E\left(mHD|B^k\right)}{n}\right) = \left(\frac{2^{19}}{2^{20}}, 0.0500\right)$, respectively. The network sizes in the vertical line increase (n increases from 5 to 20) but the same x-coordinates are the same value $\frac{1}{2}$ $\left(\frac{k}{2^n}\right.$ is fixed and k increases from 2^4 to $\left.2^{19}\right)$, where the normalized average mHD, $\frac{E\left(mHD|B^k\right)}{n}$, decreases from 0.2098 to 0.0500. Following other dotted vertical lines we have the same property.

Supplementary Figure 7

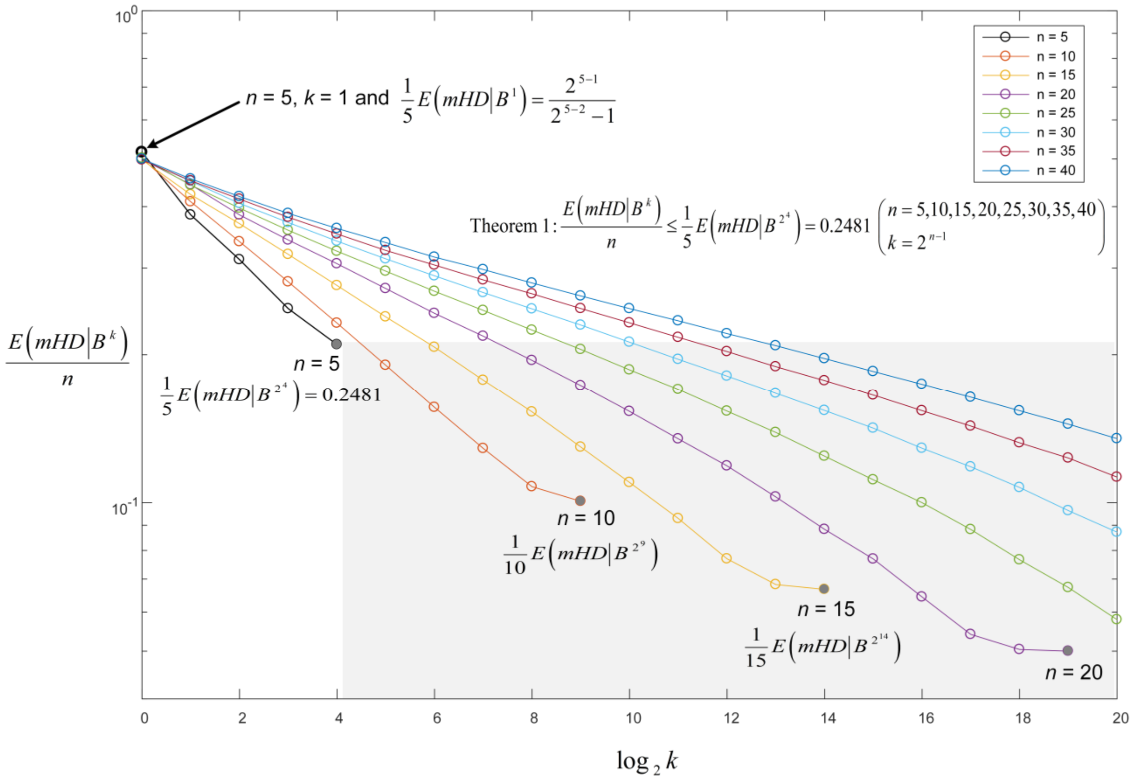

Supplementary Figure 7. Upper bound of normalized average mHD. A subset B^k of the state space Ω of a given network X of n nodes denotes a desired basin, where the positive integer k denotes the size of B^k. Symbol $E\left(mHD\middle|B^k\right)$ denotes the average mHD of undesired states to B^k. The sequence $\left\{\frac{E\left(mHD\middle|B^{2^{n-1}}\right)}{n}\right\}$ is a decreasing sequence due to Theorem 1 and Supplementary Fig. 6. The four filled circles in the grey box denote consecutive elements such that $\frac{E\left(mHD\middle|B^{2^{14}}\right)}{15} < \frac{E\left(mHD\middle|B^{2^{9}}\right)}{10} < \frac{E\left(mHD\middle|B^{2^{4}}\right)}{5}$. In addition, the grey box shows that $\frac{E\left(mHD\middle|B^{2^{\theta}}\right)}{15} < \frac{E\left(mHD\middle|B^{2^{\ell}}\right)}{10} < \frac{E\left(mHD\middle|B^{2^{4}}\right)}{5}$ for $\theta = 12,13,14$ and $\ell = 5,6,7,8$, which are not proved in Theorem 1. Value $\frac{1}{5}E\left(mHD\middle|B^1\right) = \frac{2^{5-1}}{2^{5-2}-1}$ is an upper bound of all $\frac{E\left(mHD\middle|B^k\right)}{n}\left(n = 5,10,\cdots,35,40, k = 2^{n-1}\right)$ as shown in Remark 4.

Supplementary Figure 8

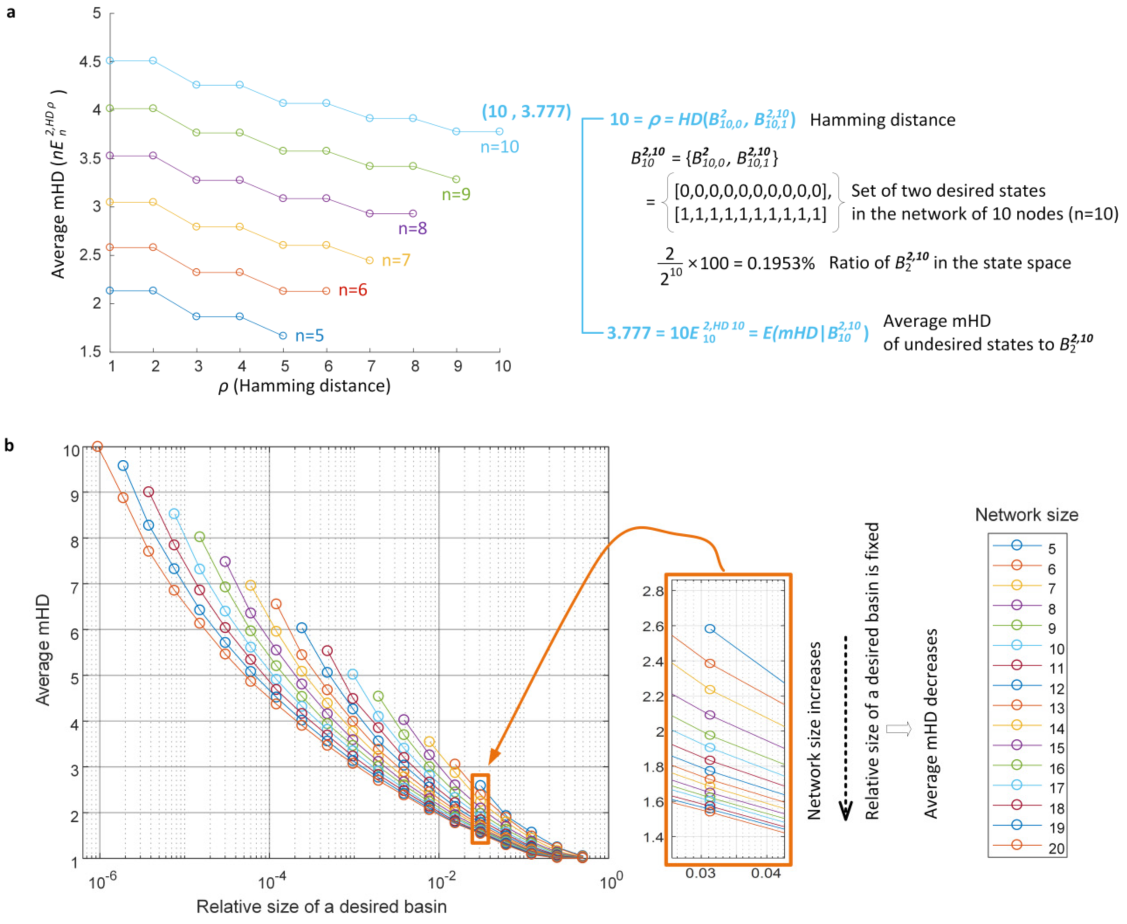

Supplementary Figure 8. Two factors to reduce mHD. a A subset $B_n^{2,\rho}=\{B_{n,0}^2,B_{n,1}^{2,\rho}\}$ of the state space Ω of a given network X of n nodes denotes a desired basin, where the state values of nodes in $B_{n,0}^2=(0,\cdots,0)$ are zero and $B_{n,1}^{2,\rho}=(0,\cdots,0,1,\cdots,1)$ has value 0 of all nodes except for the last ρ nodes $(1\le\rho\le 10)$. Note that superscripts 2 and ρ in $B_n^{2,\rho}$ denote the number of states in $B_n^{2,\rho}$ and the HD between $B_{n,0}^2$ and $B_{n,1}^{2,\rho}$, respectively. An undesired state is defined as a state contained in $\Omega-B_n^{2,\rho}$. Symbol $E\left(mHD\middle|B_n^{2,\rho}\right)$ denotes the average mHD of

undesired states to $B_n^{2,\rho}$. For simplicity symbol $nE_n^{2,HD1}$ is used instead of average mHD $E\left(mHD \middle| B_n^{2,\rho}\right)$. Every desired basin used here consists of two states. The larger the HD of two states in a desired basin is, the smaller average mHD is, which can be expressed as $nE_n^{2,HD\,\rho} \geq nE_n^{2,HD\,\rho+1}$. The right shows that even if the relative size of a basin is extremely small (0.1953%), the average mHD is less than 3.8. **b** Average mHD decreases as the network size increases and the relative size of a desired basin is fixed.

Supplementary Figure 9

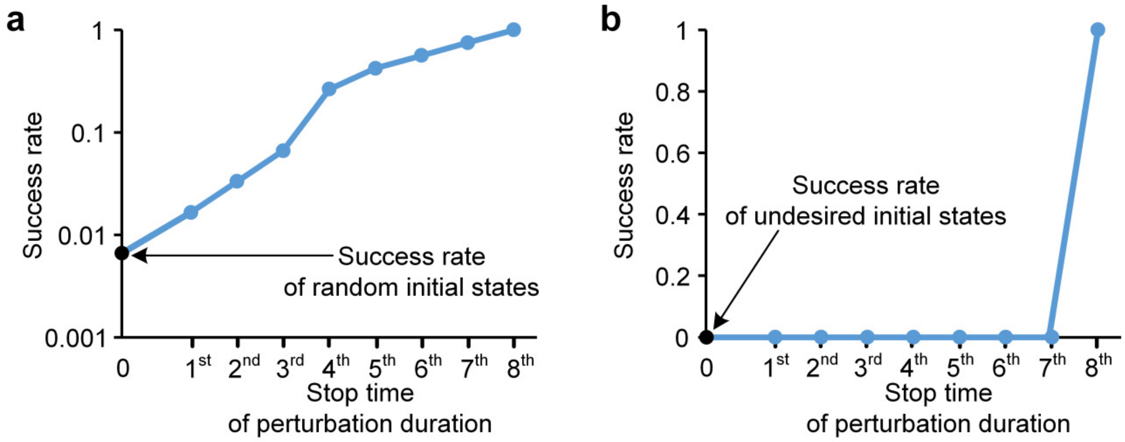

Supplementary Figure 9. Success rate of persistent perturbation depending on stop time of perturbation duration and initial states of the MAPK33 network. a Success rate for 2^{33} randomly selected initial states. The black circle on the y-axis denotes the ratio of states converging to the desired attractor in the main text and the number 0 in the x-axis indicates no perturbation. Replacing the values of BCL2, ERK, FOXO3, P21 and P53 in all the 2^{33} randomly selected states with (BCL2, ERK, FOXO3, P21, P53) = (0, 0, 1, 1, 1) gives perturbed states, which are referred to as the "1st perturbed random states". Substituting one of the 1st perturbed random states into the update rules (Supplementary Data 4) as an initial state at time step 1 gives a state of MAPK33 at time step 2, which is referred to as a "2nd state". Similarly, "nth states" are defined. The ratio of the 1st perturbed random states converging to the desired attractor in the main

text is the "success rate" at the 1st stop time of perturbation duration. Note that no persistent perturbation is applied for the calculation of success rate. Similarly, replacing the values of BCL2, ERK, FOXO3, P21 and P53 in all the 1st states with (BCL2, ERK, FOXO3, P21, P53) = (0, 0, 1, 1, 1) gives perturbed states, which are referred to as the "2nd perturbed random states" and the ratio of the 2nd perturbed random states converging to the desired attractor is the success rate at the 2nd stop time of perturbation duration. At the 8th stop time of perturbation duration the success rate becomes 1. **b** Success rate for 1,310,720 initial states of which collection is the basin of the undesired attractor in the main text. The meanings of symbols and terms in **b** are equal to those in **a**. The difference is that all basin states of the undesired attractor are used in **b** as initial states instead of 2^{33} randomly selected initial states used in **a**. Since all the initial states are undesired basin states, the ratio of all the initial states converging to the desired attractor is equal to 0 (black circle). When the stop time increases up to the 7th stop time, states are getting close to the desired attractor (this data is not shown) but do not converge to the desired attractor.

Supplementary Figure 10

a

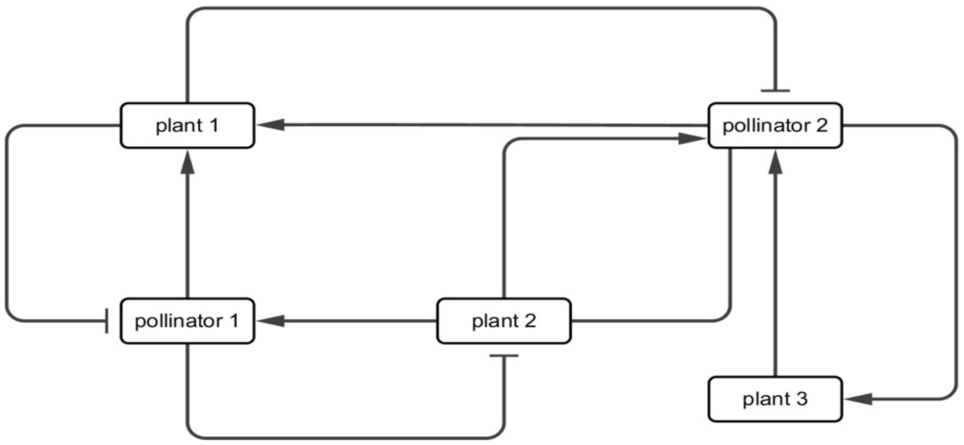

b

Node: plant 1 (pl1), plant 2 (pl2), plant 3 (pl3), pollinator 1 (po1), pollinator 2 (po2).

Attractor (attr1)
Undesired

pl1	pl2	pl3	po1	po2
**0**	**0**	**0**	**0**	**0**

Attractor (attr2)
Unrealistic

pl1	pl2	pl3	po1	po2
0	0	0	0	1
1	1	1	0	0

Attractor (attr3)
Desired

pl1	pl2	pl3	po1	po2
**1**	**1**	**1**	**0**	**1**

Undesired state	basin of attr1				
1	**0**	**0**	**0**	**0**	**0**
2	0	0	0	1	0
3	0	0	0	1	1
4	0	1	0	0	0
5	0	1	0	1	0
6	0	1	0	1	1
7	0	1	1	0	0
8	0	1	1	1	0
9	0	1	1	1	1
10	1	0	0	0	0
11	1	0	0	1	0
12	1	0	0	1	1
13	1	0	1	0	0
14	1	0	1	1	0
15	1	0	1	1	1
16	1	1	0	0	0
17	1	1	0	1	0
18	1	1	0	1	1

State	basin of attr2				
1	0	0	0	0	1
2	0	0	1	0	0
3	0	0	1	1	0
4	0	0	1	1	1
5	0	1	0	0	1
6	0	1	1	0	1
7	1	0	0	0	1
8	1	0	1	0	1
9	1	1	0	0	1
10	1	1	1	0	0
11	1	1	1	1	0
12	1	1	1	1	1

Desired state	basin of attr3				
1	0	0	1	0	1
2	**1**	**1**	**1**	**0**	**1**

Minimum of mHDs of undesired states to the basin of attr 3 is equal to 2.

Supplementary Figure 10. A Boolean network model for plant–pollinator community assembly and application of BRC. a The interaction network of three

plants species (plant 1, plant 2 and plant 3) and two pollinators species (pollinator 1 and pollinator 2)[1]. Arrows and bar-headed lines in the network represent activation and inhibition, respectively. **b** Restoration of the desired ecological state. Symbol "attr1" is an undesired attractor due to no species in attr1. Symbol "attr2" is an unrealistic attractor due to its cyclic property[1]. Symbol "attr3" is assumed to be a desired attractor. The control target species to restore the desired ecological state (attr3) from the absence of all species (attr1) are plant 3 and pollinator 2.

Supplementary Figure 11

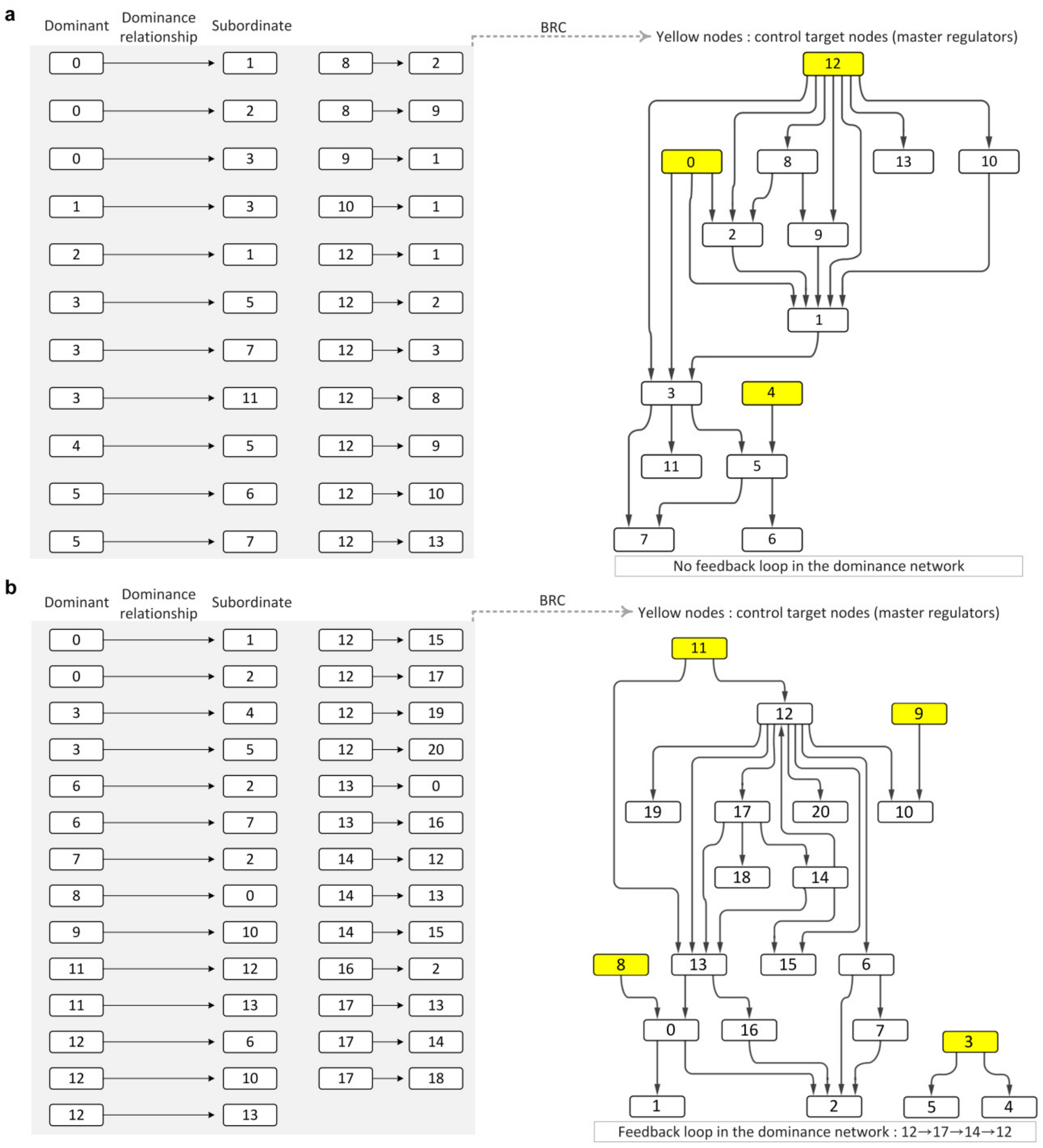

Supplementary Figure 11. Dominance interaction networks in the social wasp *Ropalidia marginata* and application of BRC. a Role of control target nodes in a dominance interaction network with no feedback loop. The colony of *R. marginata* is of size 14. Arrows on the left side denote the dominance relationship between dominants

and subordinates[2]. Each number in a box denotes a wasp. The right shows that the dominant network has the structure of feed-forward loop without a feedback loop. For the application of BRC, the Boolean logic OR is used for all the interactions. The state of each node in a desired attractor is assumed to be value 1 and undesired states are all states except basin states of the desired attractor. The yellow nodes denote control target nodes, which are located at the tops of dominant relationships and so can be called "master regulators". There is no master regulator except for the control target nodes. **b** Role of control target nodes in a dominance interaction network with a feedback loop. The colony of *R. marginata* is of size 21. Arrows on the left side denote the dominance relationship between dominants and subordinates[2]. The right shows that the dominant network has the structure of feed-forward loop except for the feedback loop 12→17→14→12. For the application of BRC, the OR logic is used for all the interactions. The state of each node in a desired attractor is assumed to be value 1 and undesired states are all states except basin states of the desired attractor. The yellow nodes denote control target nodes, which are located at the tops of dominant relationships and so can be called "master regulators". There is no master regulator except for the control target nodes.

Supplementary Figure 12

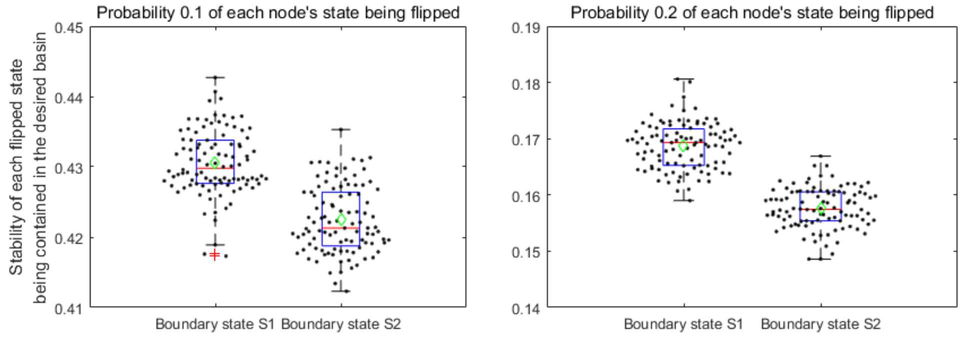

Supplementary Figure 12. Stability comparison of two boundary states (S1 and S2). CACC21 network in Fig. 2 and Supplementary Data 2 are used in the boxplots. A cancer attractor state (att1 in Supplementary Data 2) has two boundary states (S1 and S2) which converge to a desired attractor state (att4 in Supplementary Data 2). Each node's state in S1 was flipped 10,000 times with probability 0.1 (left). As a result, a probability of the flipped state being contained in the desired basin was calculated (referred to as stability). Repeating the calculation of stability 100 times, we obtained 100 stabilities of S1 and represented them as black dots in the first column on the left side (a green diamond for average stability). Similarly, we obtained 100 stabilities of S2 in the second column on the left side. When comparing box plots of S1 and S2, boundary state S1 can be considered more stable than S2. In the case of probability 0.2 (right), we obtained the same result that S1 can be considered more stable than S2.

Reference

1. Campbell C, Yang S, Albert R, Shea K. A network model for plant-pollinator community assembly. *Proc Natl Acad Sci U S A* **108**, 197-202 (2011).

2. Nandi AK, Sumana A, Bhattacharya K. Social insect colony as a biological regulatory system: modelling information flow in dominance networks. *J R Soc Interface* **11**, (2014).
